# Supplementary material for: HDAC1 dysregulation induces aberrant cell cycle and DNA damage in progress of TDP‐43 proteinopathies
Source: EMBO Mol Med. 2020 May 25;12(6):e10622. doi: 10.15252/emmm.201910622 (PMC7278561; doi:10.15252/emmm.201910622)
Supplement: Supplementary file 1 — Appendix [file EMMM-12-e10622-s001.pdf]

HDAC1 Dysregulation Induces Aberrant Cell Cycle and DNA Damage in Progress of TDP-43 Proteinopathies

Cheng-Chun Wu, Lee-Way Jin, I-Fang Wang, Wei-Yen Wei, Pei-Chuan Ho, Yu-Chih Liu and Kuen-Jer Tsai

<u>Appendix</u>

Table of contents…………..…………..…………..…………..………….……..page 2

Appendix Figure S1…………..…………..…………..………………..……..page 3

Appendix Supplementary Method…………..…………..……..…..…………page 4

Appendix Table S1……..…………..………………………………….……..…page 5

Appendix Fig. S1

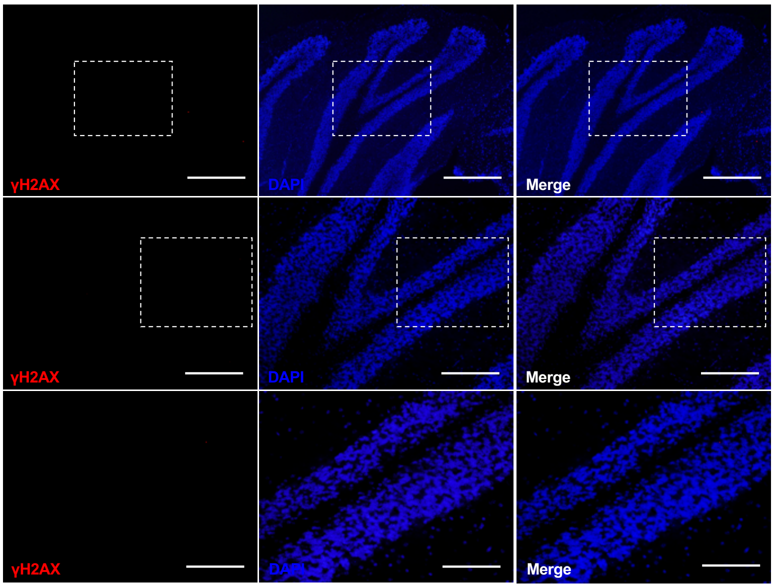

Appendix Figure S1. Staining of γH2AX and DAPI in the cerebellum of 12-month-old FTLD-TDP Tg mice.

Representative IF staining of γH2AX and DAPI in the cerebellum of 12-month-old FTLD-TDP Tg mice. At this time point, we cannot detect any γH2AX immunoreactive cells. Upper graph, scale bar: 500 µm. The circled area is emphasized for showing the distribution of immunoreactivity in cell subregions. Middle graph, scale bar: 200 µm, lower graph, scale bar : 100 µm. n= 4 sections per mouse, N = 5 mice per group.

Appendix Supplementary Method

Nuclear HDACs activity assay in cell line

For *in vitro* HDACs activity tests, SHSY5Y cells were cultured in DMEM/F12 medium (Thermo, catalog NO. 11320033) with 10% FBS (Bioindustry, 04-001-1A-US) and were induced to differentiate by neural basal medium (Thermo, catalog NO. 21103049) containing 2% of B27 supplements (Thermo, catalog NO. 17504044), 10 μM retinoid acid (Sigma, R2625), and 1% FBS for 5 days, the medium was replaced every day. For compound administration, 1, 10, 50 μM of compound 5104434 were treated into differentiated SH-SY5Y cells for 72 hour, the 5104434 contained medium medium was replaced every day. For the activity assays, the compound 5104434 treated cells were subjected to nuclear extraction following the manual instructions. The activity of HDAC1, 2, 3, 8 were evaluated by using the activity assay kit (Enzo Life Sciences, BML-AK500-0001, BML-AK512, BML-AK531, BML-AK518), total 30 μg of nuclear protein from each sample was inputted for the assay.

Appendix Table 1. List of P value of figure 1C; 6B; 7D and EV4

Fig. 1C

PCR

WT V.S. Tg		p-value	
	E2F1	< 0.0001	****
	Cyclin E	0.025439821	*
	PCNA	0.013657367	*
	p21	0.000533315	***
Western blot			
WT V.S. Tg		p-value	
	E2F1	0.004175469	**
	Cyclin A	0.036820881	*
	PCNA	0.00323307	**
	p21	< 0.0001	****
	γH2AX	0.001980008	**

Fig. 6B

		p-value	
trial3	Tg+Vehicle vs. WT+Vehicle	0.0004	***
trial4	Tg+Vehicle vs. WT+Vehicle	<0.0001	****
	Tg+Vehicle vs. Tg+5104434	0.0199	#
trial5	Tg+Vehicle vs. WT+Vehicle	<0.0001	****
	Tg+Vehicle vs. Tg+5104434	< 0.0001	####
trial6	Tg+Vehicle vs. WT+Vehicle	<0.0001	****
	Tg+Vehicle vs. Tg+5104434	< 0.0001	####

Fig. 7D

WT V.S. Tg		p-value	
	E2F1	< 0.0001	****
	PCNA	0.0019	**
	p21	< 0.0001	****
	γH2AX	< 0.0001	****
Tg V.S. Tg+5104434			
	E2F1	< 0.0001	****
	PCNA	0.0972	
	p21	< 0.0001	****
	γH2AX	0.0005	***

Fig. EV4B

		P value	
trial3	*WT_veh vs Tg_Veh*	<0.0001	****
trial4	*WT_veh vs Tg_Veh*	<0.0001	****
trial5	*WT_veh vs Tg_Veh*	<0.0001	****
trial6	*WT_veh vs Tg_Veh*	<0.0001	****
trial4	*Tg_veh vs Tg_6mg*	0.0438	#

trial5	*Tg_veh vs Tg_6mg*	0.0381	#
trial6	*Tg_veh vs Tg_6mg*	0.0001	###
trial5	*Tg_veh vs Tg_30mg*	0.0489	@
trial6	*Tg_veh vs Tg_30mg*	0.001	@@@

Fig. EV4D

EV4D-1	EV4D-1	P value	
	WT_veh vs Tg_Veh	<0.0001	****
	Tg_veh vs Tg_6mg	0.0041	**
	Tg_veh vs Tg_30mg	0.0006	***
EV4D-2	EV4D-2	P value	
	WT_veh vs Tg_Veh	<0.0001	****
	Tg_veh vs Tg_6mg	0.0355	*
	Tg_veh vs Tg_30mg	0.0091	**

Fig. EV4E

		P value	
	WT_veh vs Tg_Veh	0.0002	***
	Tg_veh vs Tg_6mg	0.0119	*
	Tg_veh vs Tg_30mg	0.0055	**
