# Supplementary material for: HDAC1 dysregulation induces aberrant cell cycle and DNA damage in progress of TDP‐43 proteinopathies
Source: EMBO Mol Med. 2020 May 25;12(6):e10622. doi: 10.15252/emmm.201910622 (PMC7278561; doi:10.15252/emmm.201910622)
Supplement: Supplementary file 2 — Expanded View Figures PDF [file EMMM-12-e10622-s002.pdf]

Expanded View Figures

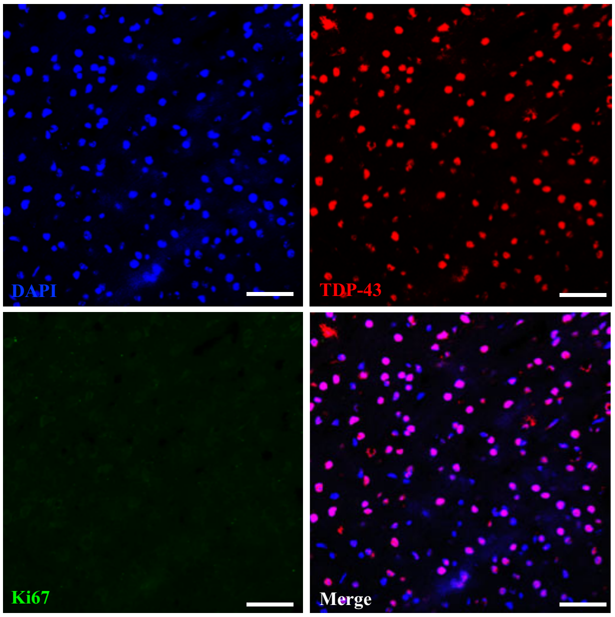

Figure EV1. Co-staining of TDP-43 and cell cycle marker Ki67 in the brain of 2-month-old FTLD-TDP Tg mice.

Representative IF staining of Ki67, TDP-43, and DAPI in the brain region of frontal cortices of 2-month-old FTLD-TDP Tg mice. At this time point, we cannot detect any Ki67 immunoreactive cells, and most of the TDP-43 remains inside the nucleus without mislocalization. Scale bar: 50 μm. n = 4 sections per mouse, N = 5 mice per group.

Source data are available online for this figure.

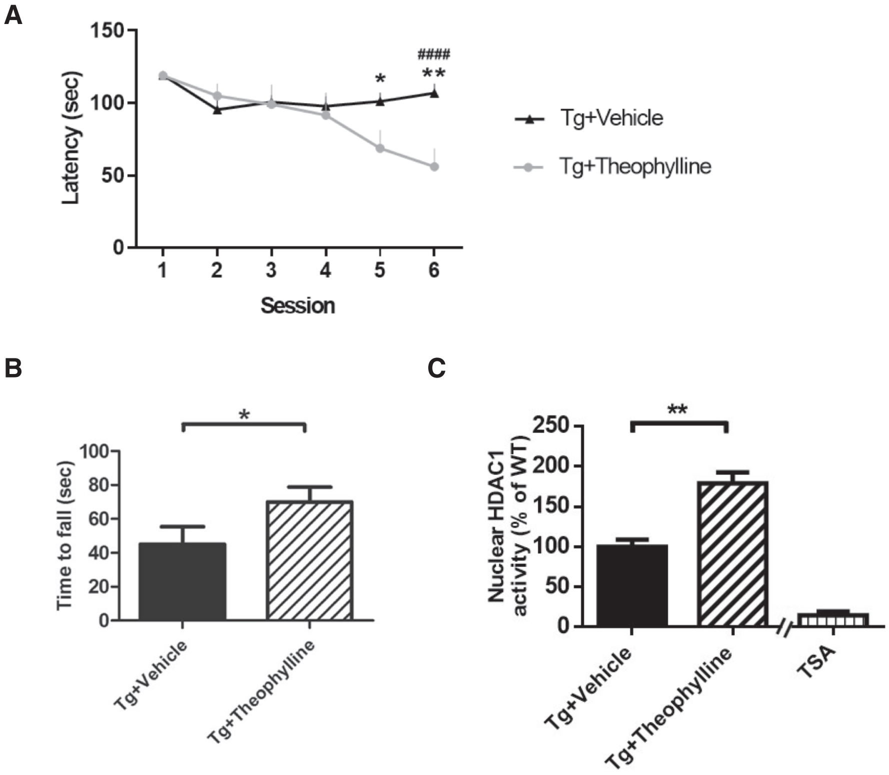

Figure EV2. Theophylline shows therapeutic potential to ameliorate cognitive and motor functions by recovery of class 1 HDACs activity.

- A Escape latency of mice in the Morris water maze task following 2 months of HDAC1 activator treatment. N = 5 mice per group. *P = 0.0469 (5th session)/**P = 0.007 or #### P < 0.0001 (6th session) by multiple comparison. * Tg+Theophylline mice versus Tg+Vehicle mice, # Tg+5104434 mice versus Tg+Vehicle mice.
- B Scores of mice in the rotarod test. N = 5 mice per group. *P = 0.0371 by Student's t-test.
- C Nuclear HDAC1 activity in mice following 2 months of theophylline treatment. N = 5 mice per group. **P = 0.0013 by Student's t-test. TSA: nuclear extracts that were treated with Trichostatin A (TSA, an HDAC inhibitor) as a negative control for HDAC1-transferred fluorescent activity during the HDAC1 activity assay.

Data information: All data are presented as mean ± SEM.

 © 2020 The Authors

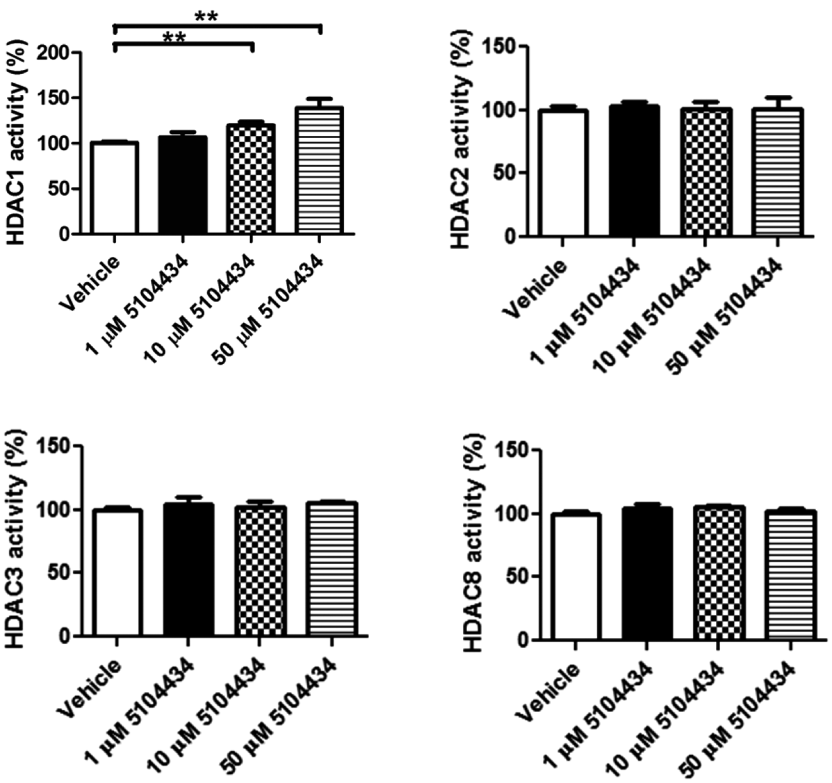

Figure EV3. Compound 5104434 specifically promotes HDAC1 activity in a dose-dependent manner but not in other class 1 members.
One, 10, and 50 μM of compound 5104434 were administrated in cultured SH-SY5Y cells for 72 h to detect HDACs activity. Ten μM of compound 5104434 increased 19.6 ± 4.1% of HDAC1 activity, 50 μM of compound 5104434 increased 39 ± 9.8% of HDAC1 activity, and only HDAC1 activity was altered following the dose-dependent assessment. $N = 5$ independent experiments. All data are presented as mean ± SEM, ** indicates $P = 0.0079$ by Mann-Whitney test.

© 2020 The Authors

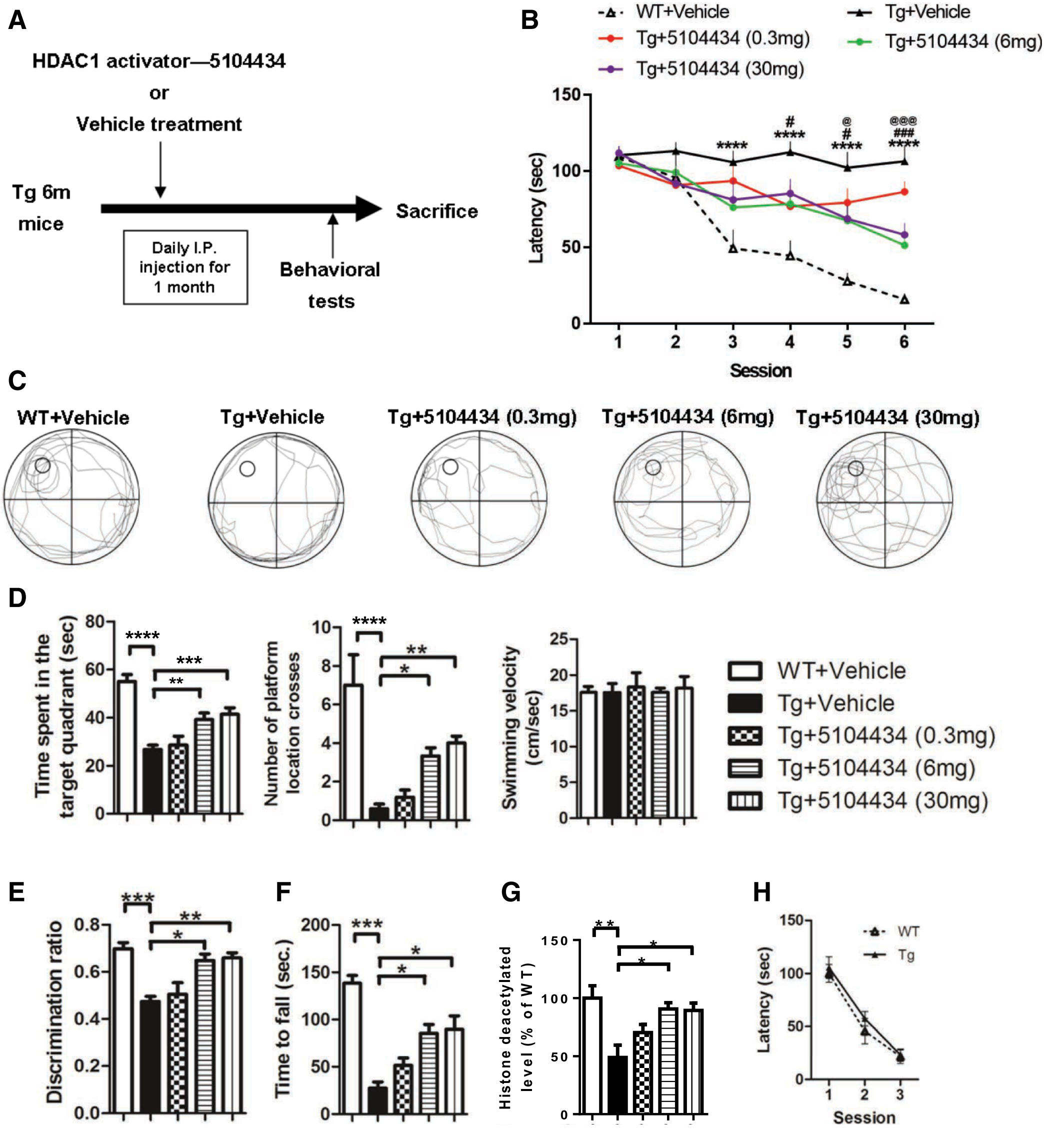

Figure EV4. Therapeutic potential and dose evaluation of compound 5104434 for treating cognitive and motor deficits in FTLD-TDP Tg mice.

A Schematic diagram shows the protocol used for HDAC1 activator compound 5104434 treatment and behavioral tests. Three doses were tested for 1 month to determine the optimum therapeutic dose.

B Escape latency of mice in Morris water maze task. N = 6 mice per group, *WT+Vehicle mice versus Tg+Vehicle mice, #Tg+5104434 (6 mg) mice versus Tg+Vehicle mice. @Tg+5104434 (30 mg) mice versus Tg+Vehicle mice. Statistical analysis by two-way ANOVA with Bonferroni's multiple comparisons. #/@$P < 0.05$, ###/@@@$P \leq 0.001$ and ****$P \leq 0.0001$. Exact P values are shown in Appendix Table S1.

C Representative searching path of mice in the probe test.

D Scores of mice as per the number of times the hidden platform was crossed, time spent searching in the target quadrant, and the velocity of swimming in the probe test at 24 h after escape training. N = 6 mice per group. Statistical analysis by one-way ANOVA with Tukey's multiple comparisons. *$P < 0.05$, **$P \leq 0.01$; ***$P \leq 0.001$ and ****$P \leq 0.0001$. Exact P values are shown in Appendix Table S1.

E Scores of the discrimination index in the novel object recognition test. N = 6 mice per group. Statistical analysis by one-way ANOVA with Tukey's multiple comparisons. *$P < 0.05$, **$P \leq 0.01$; ***$P \leq 0.001$. Exact P values are shown in Appendix Table S1.

F Scores of mice in the rotarod test. N = 6 mice per group. *P = 0.0482 (6 mg)/0.0392 (30 mg), ***P = 0.0001 by multiple comparison.

G Nuclear HDAC1 activity in mice following 1 month of 5104434 treatment. N = 6 mice per group. *P = 0.0117 (6 mg)/0.0151 (30 mg), **P = 0.0016 by multiple comparison.

H Visible platform test in WT and FTLD-TDP Tg mice. N=6 mice per group.

Data information: All data are presented as mean ± SEM.

 © 2020 The Authors

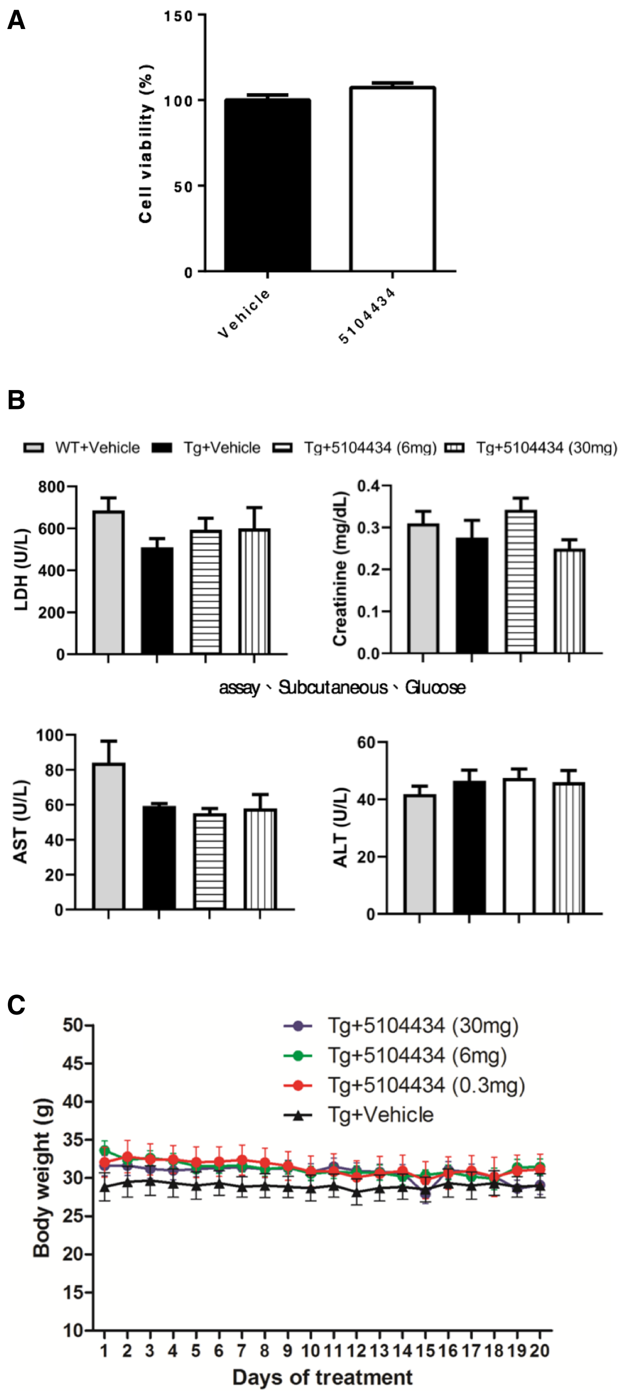

Figure EV5. Evaluation of the pharmaceutical side effects with respect to cell viability in cell model, hepatotoxicity, renal toxicity, and body weight after 1 month of compound 5104434 treatment in animal model.

A Cell viability was detected under 5104434 treatment for 72 h in 293T cell line. *N* = 3 independent experiments.

B Evaluation of the serum chemistry profile including lactate dehydrogenase (LDH), creatinine, aspartate transaminase (AST), and alanine transaminase (ALT) in both WT and FTLD-TDP Tg mice treated with vehicle, 6 mg/kg/day, and 30 mg/kg/day of 5104434. *N* = 10, 5, 5, 15 mice in each group.

C Records of body weight in vehicle and 5104434-treated FTLD-TDP Tg mice. *N* = 5 mice per group.

Data information: All data are presented as mean ± SEM.

© 2020 The Authors
