# Supplementary figures and images for: HDAC1 dysregulation induces aberrant cell cycle and DNA damage in progress of TDP‐43 proteinopathies
Source: EMBO Mol Med. 2020 May 25;12(6):e10622. doi: 10.15252/emmm.201910622 (PMC7278561; doi:10.15252/emmm.201910622)

**Fig. EV1**

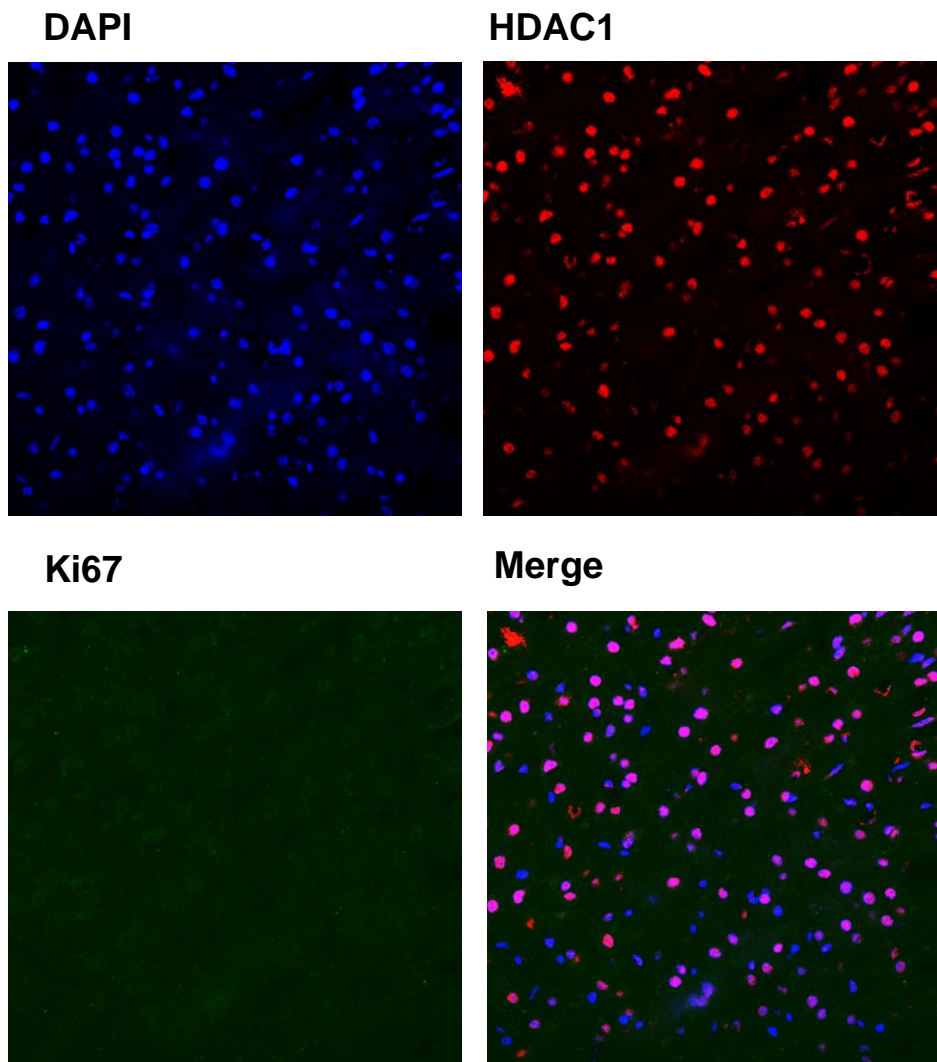

Supplement: Supplementary file 3 — Source Data for Expanded View [file EMMM-12-e10622-s010.zip › emmm201910622-sup-0010-SDataFigEV1.pdf]

**Fig. 8A**

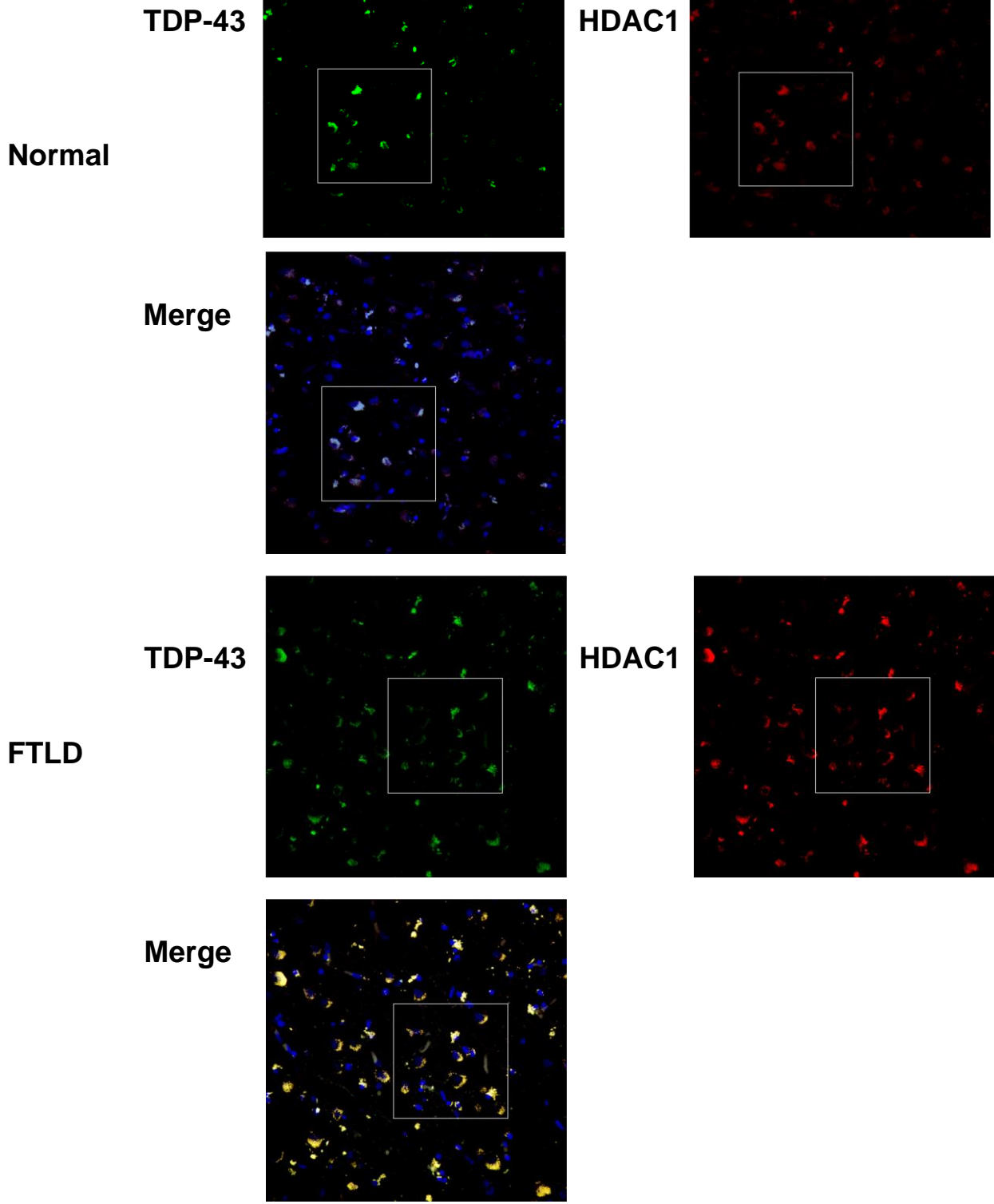

**Fig. 8D**

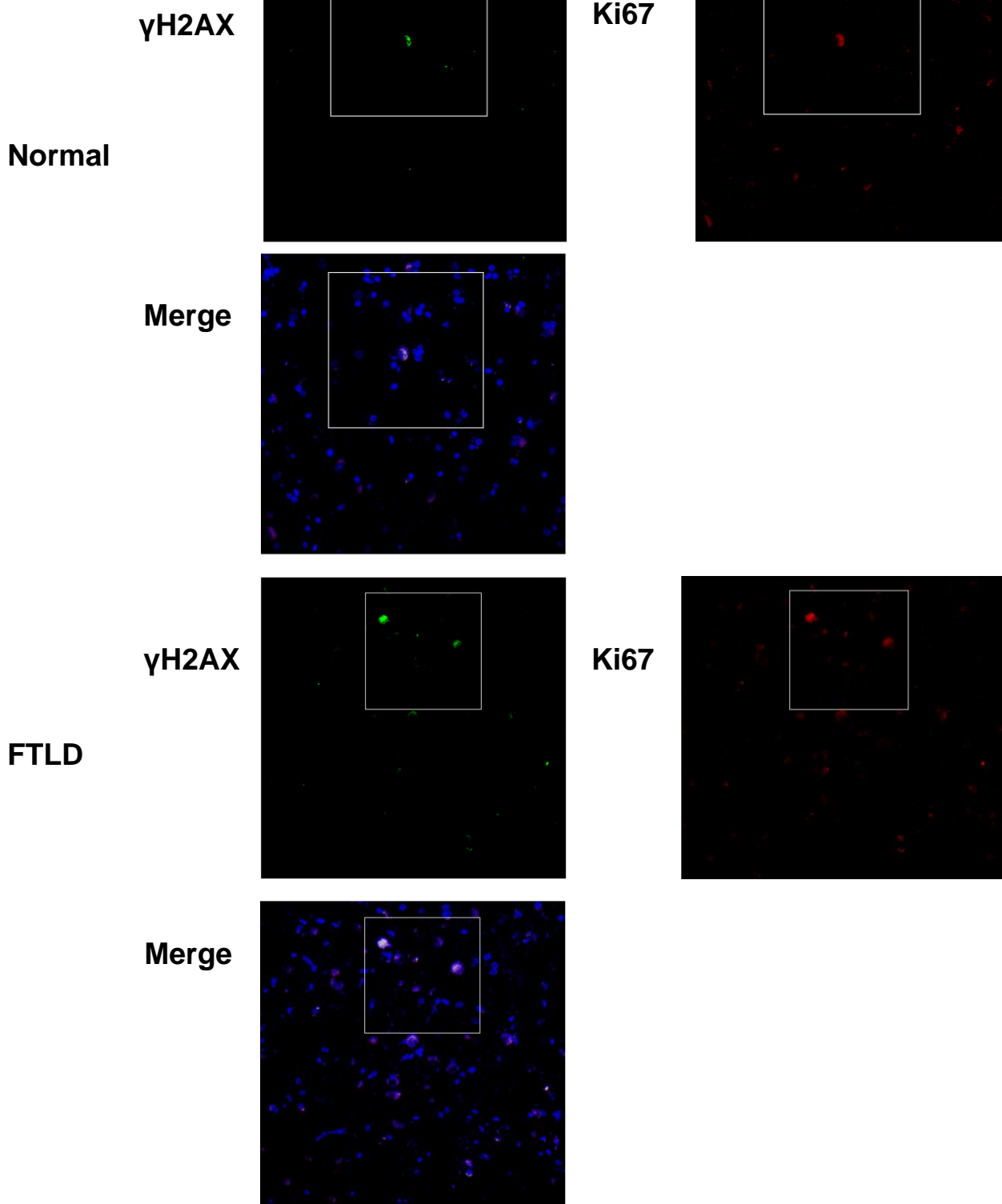

**Fig. 8G**

**HDAC1**

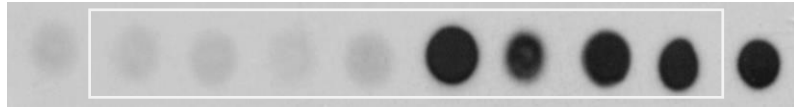

**TDP-43**

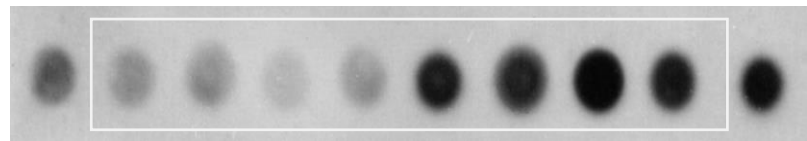

Supplement: Supplementary file 11 — Source Data for Figure 8 [file EMMM-12-e10622-s009.pdf]
