# Supplementary material for: HDAC1 dysregulation induces aberrant cell cycle and DNA damage in progress of TDP‐43 proteinopathies
Source: EMBO Mol Med. 2020 May 25;12(6):e10622. doi: 10.15252/emmm.201910622 (PMC7278561; doi:10.15252/emmm.201910622)

Fig. 1A-1

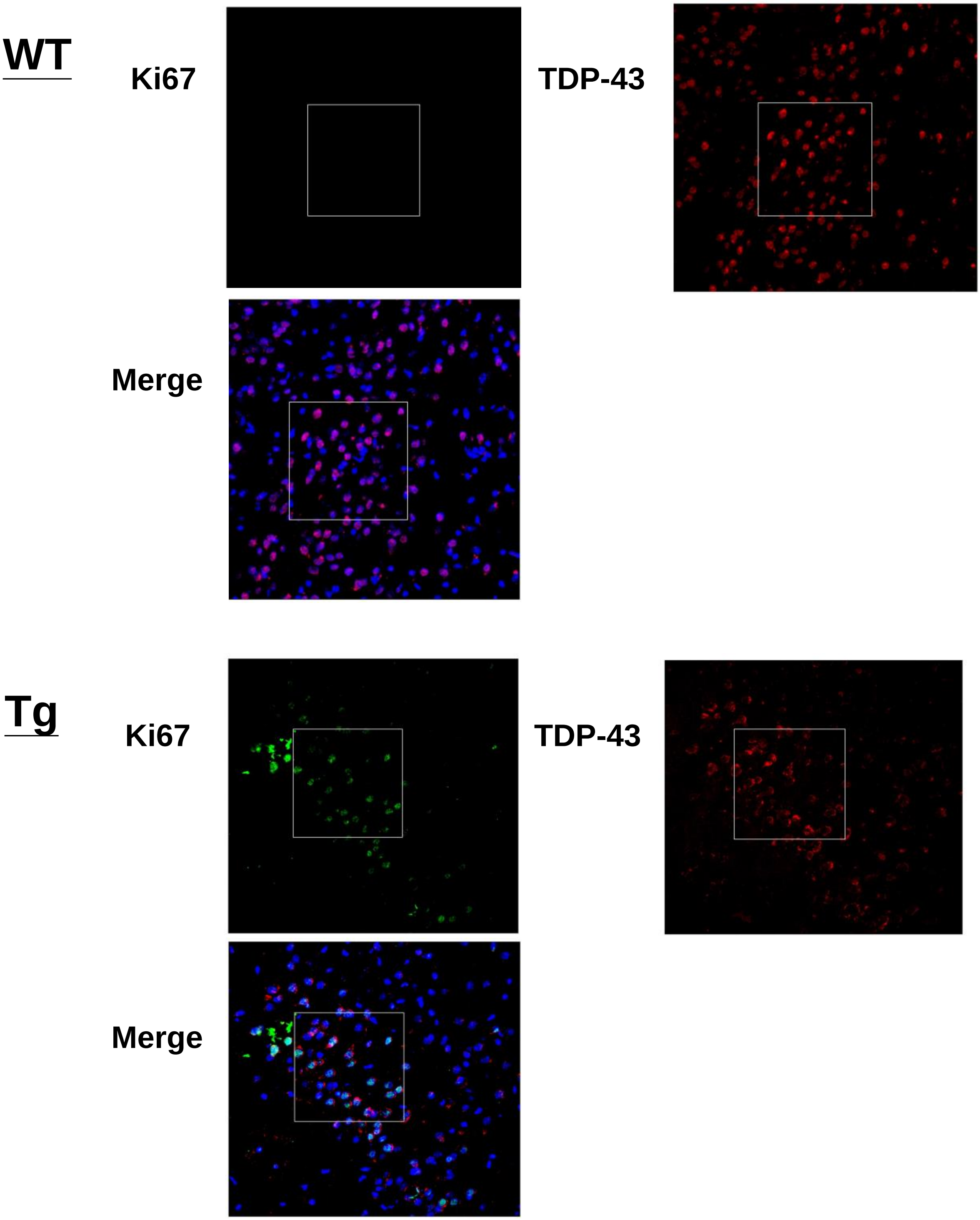

Fig. 1A-2

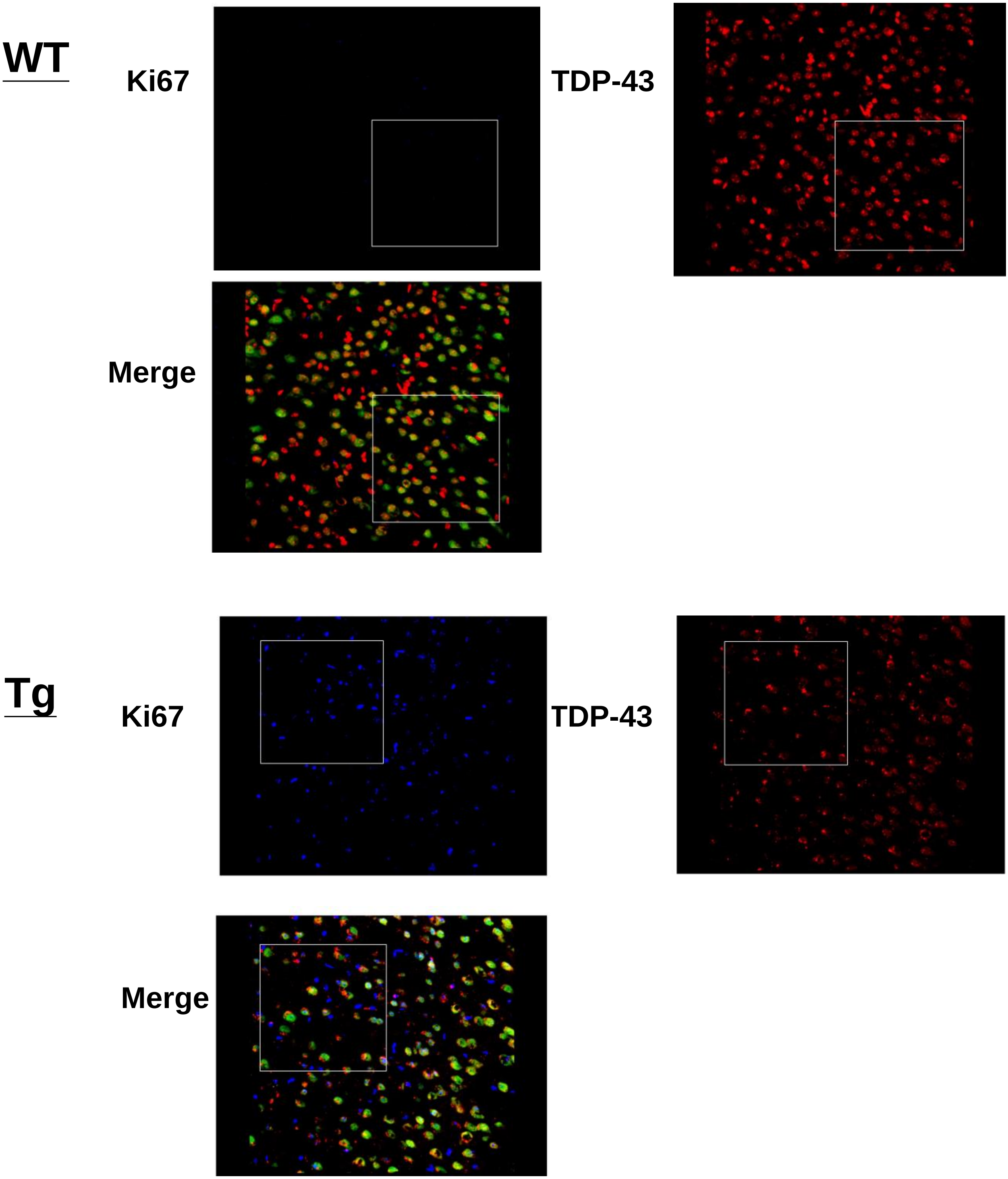

Fig. 1C

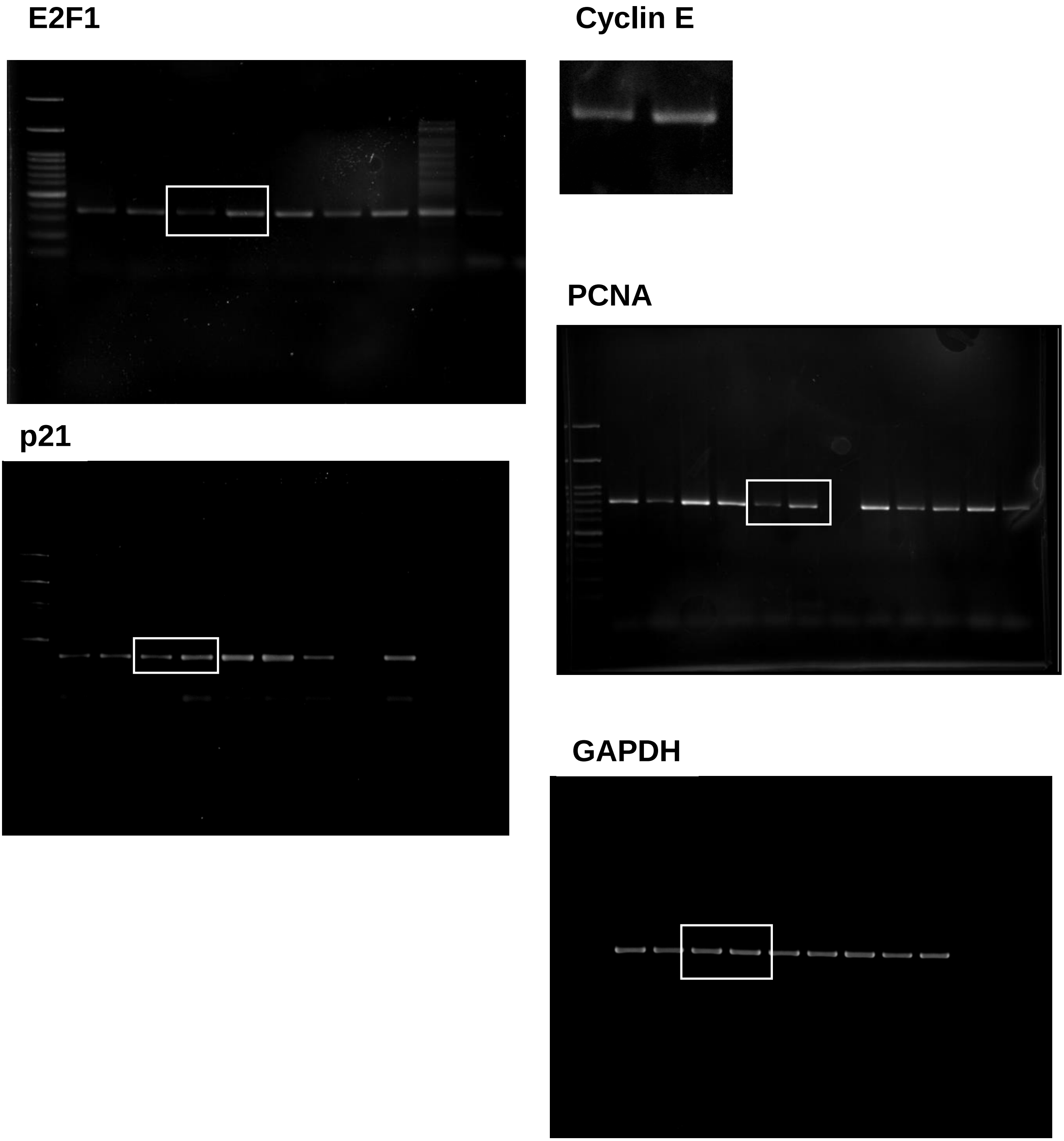

Fig. 1C

E2F1

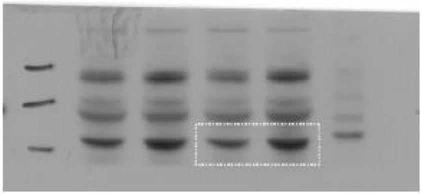

Cyclin A

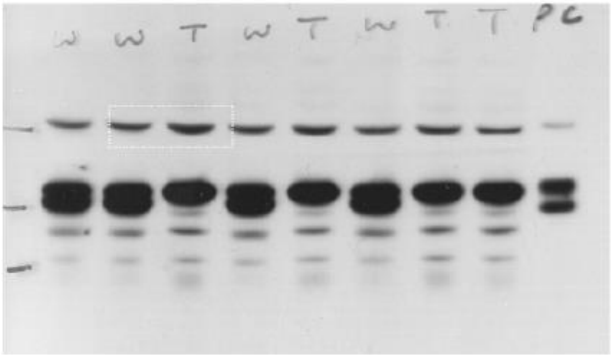

γH2AX

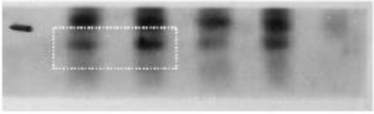

Tubulin

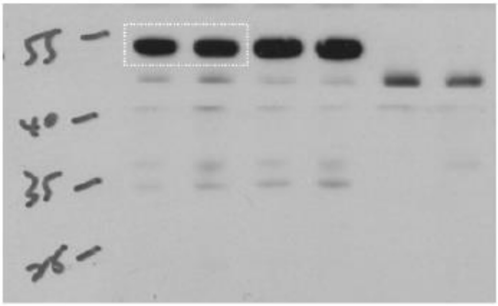

PCNA

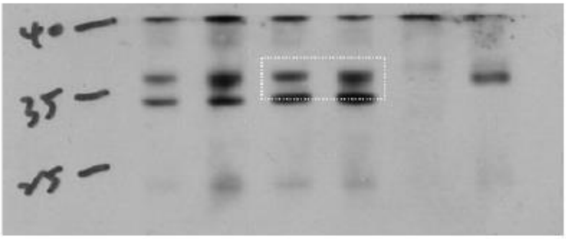

p21

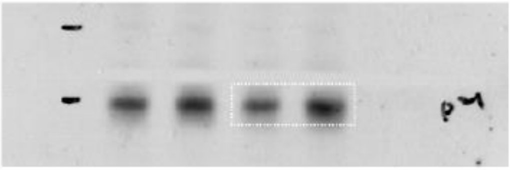

Supplement: Supplementary file 5 — Source Data for Figure 1 [file EMMM-12-e10622-s003.pdf]
