# Supplementary material for: HDAC1 dysregulation induces aberrant cell cycle and DNA damage in progress of TDP‐43 proteinopathies
Source: EMBO Mol Med. 2020 May 25;12(6):e10622. doi: 10.15252/emmm.201910622 (PMC7278561; doi:10.15252/emmm.201910622)

Fig. 2A

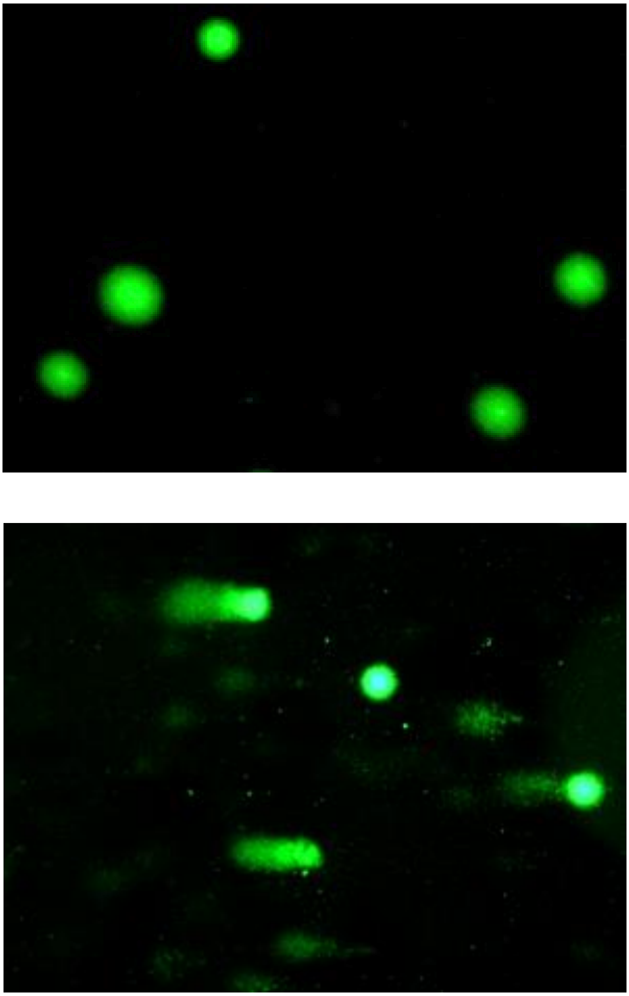

WT

Tg

Fig. 2B-1

WT

γH2AX

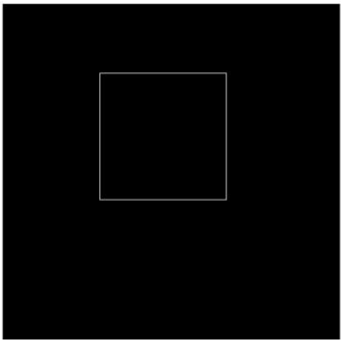

TDP-43

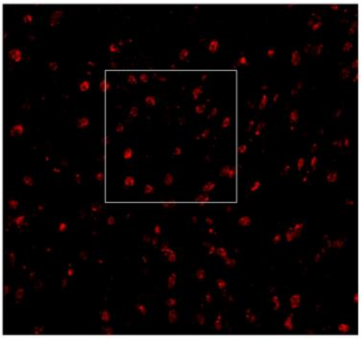

Merge

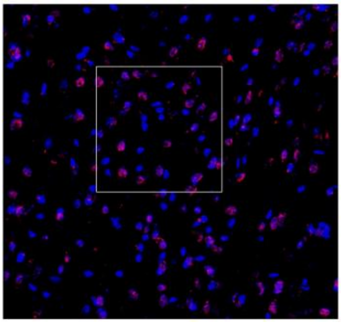

Tg

γH2AX

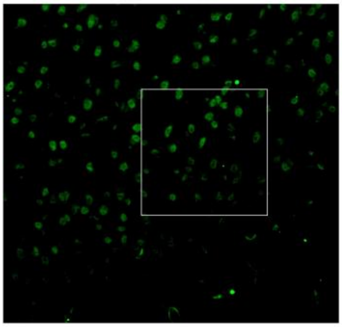

TDP-43

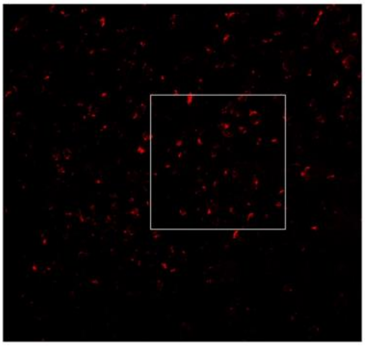

Merge

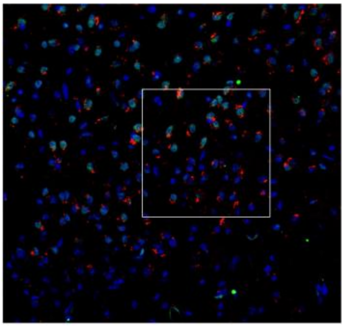

Fig. 2B-2

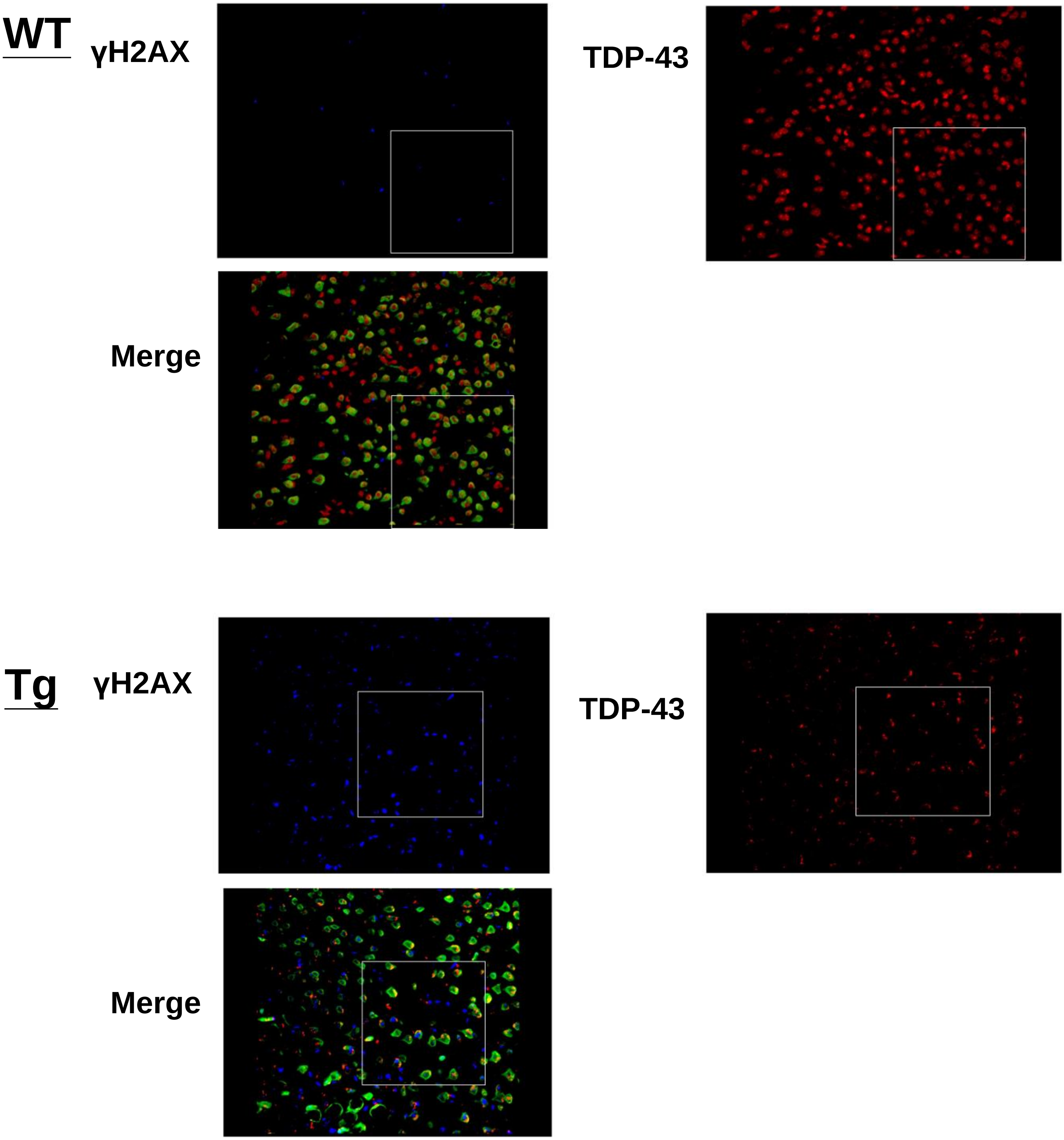

Fig. 2C-1

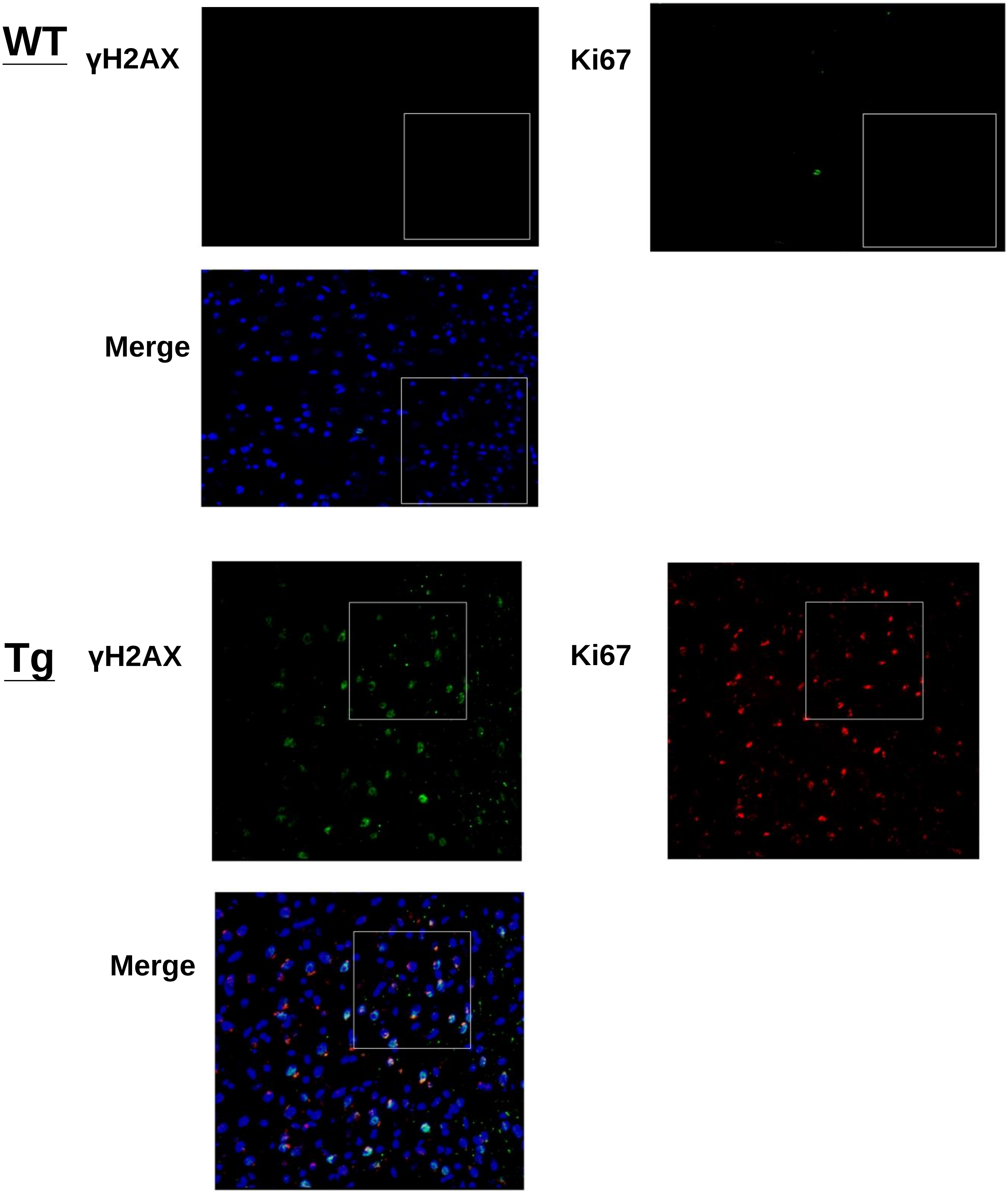

Fig. 2C-2

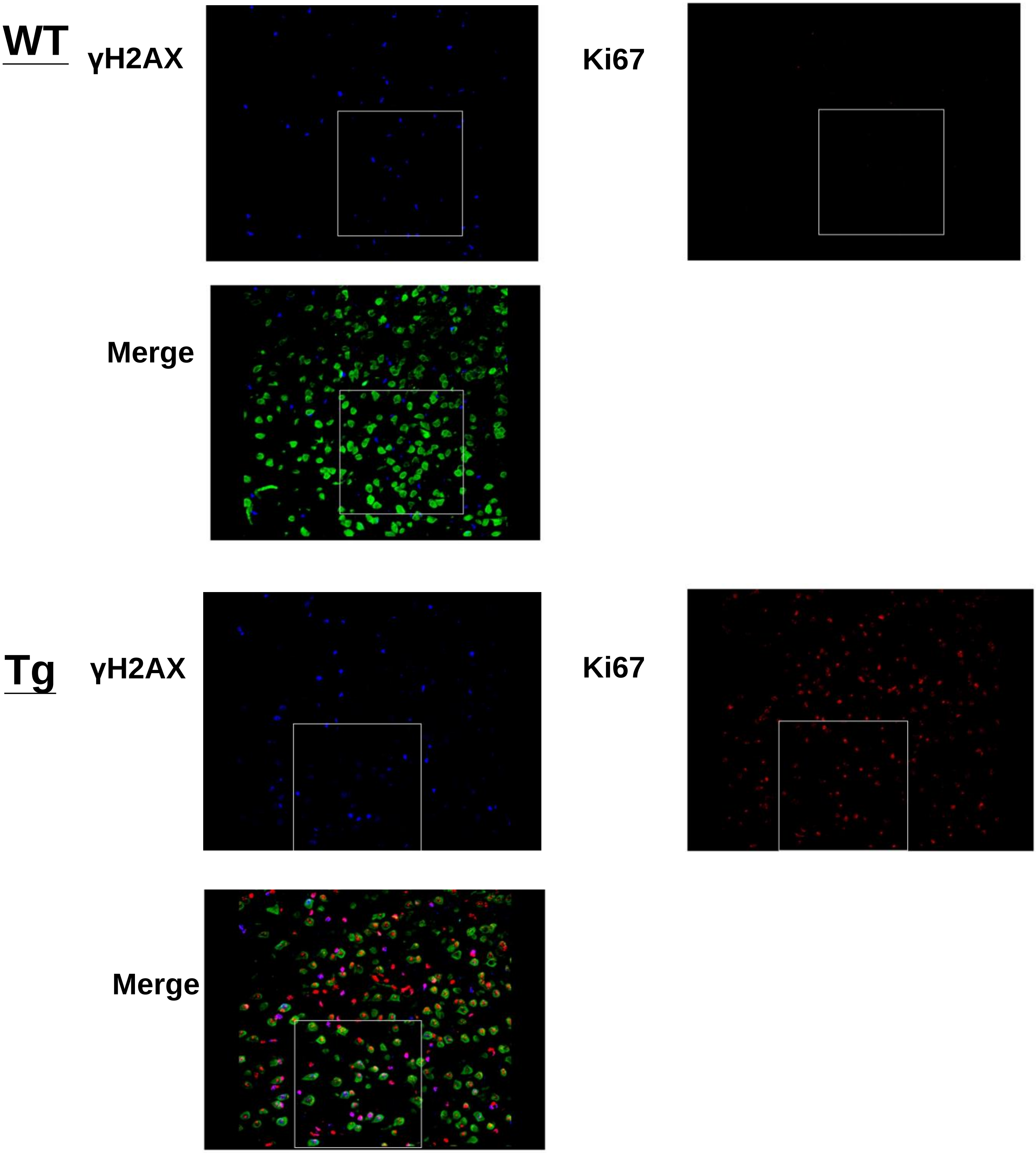

Supplement: Supplementary file 6 — Source Data for Figure 2 [file EMMM-12-e10622-s004.pdf]
