# Supplementary material for: HDAC1 dysregulation induces aberrant cell cycle and DNA damage in progress of TDP‐43 proteinopathies
Source: EMBO Mol Med. 2020 May 25;12(6):e10622. doi: 10.15252/emmm.201910622 (PMC7278561; doi:10.15252/emmm.201910622)

Fig. 3A

HDAC1

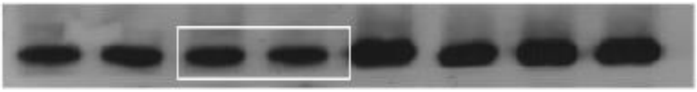

Tubulin

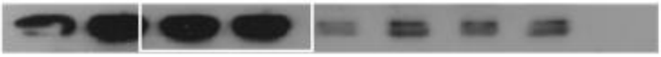

TDP-43

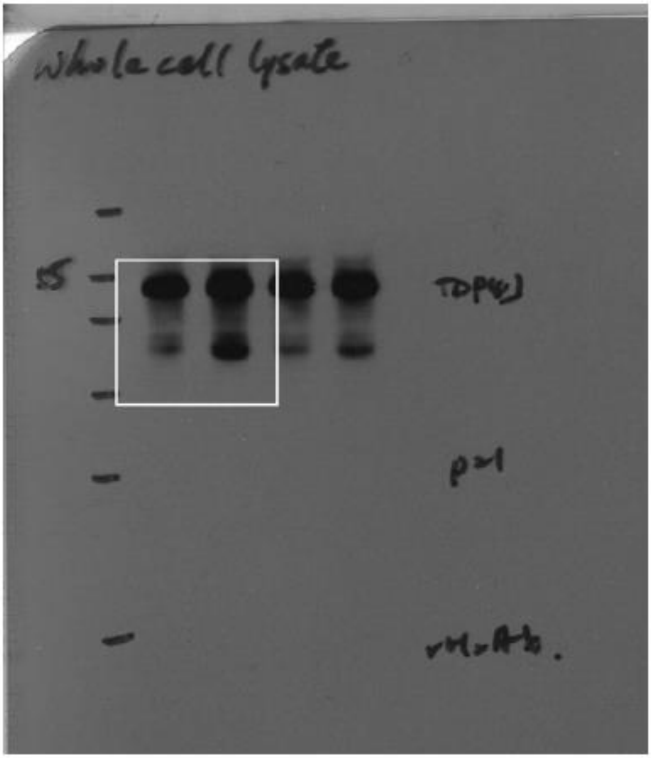

Tubulin

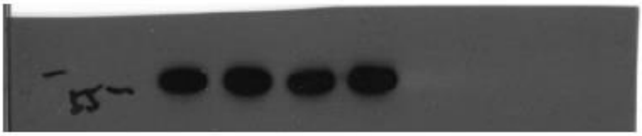

Fig. 3B

HDAC1

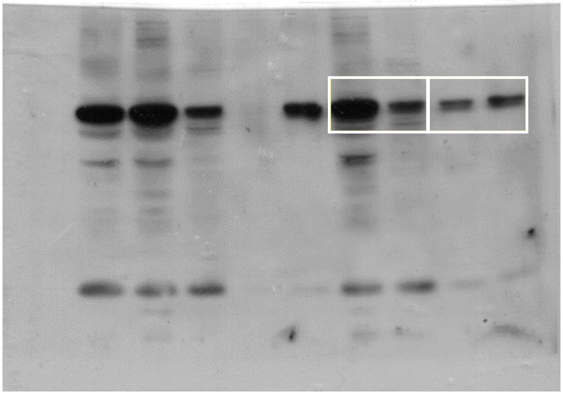

Tubulin

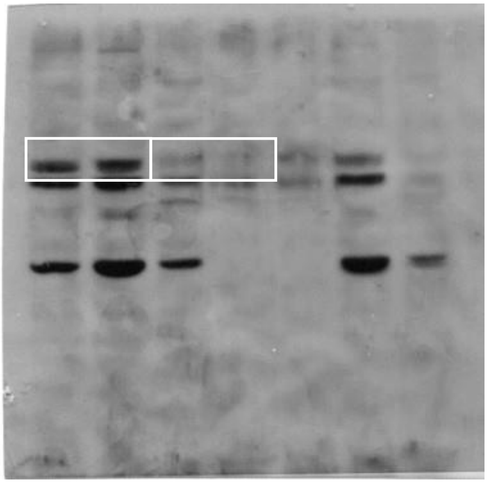

Lamin A

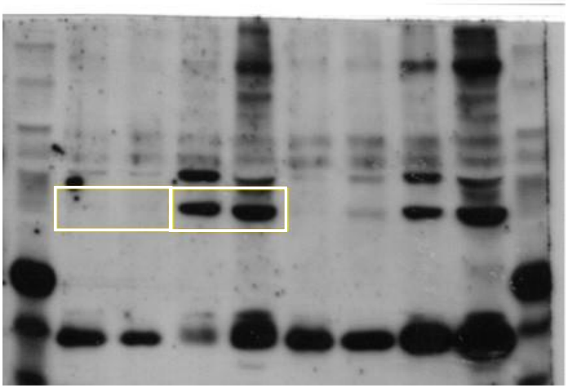

TDP-43

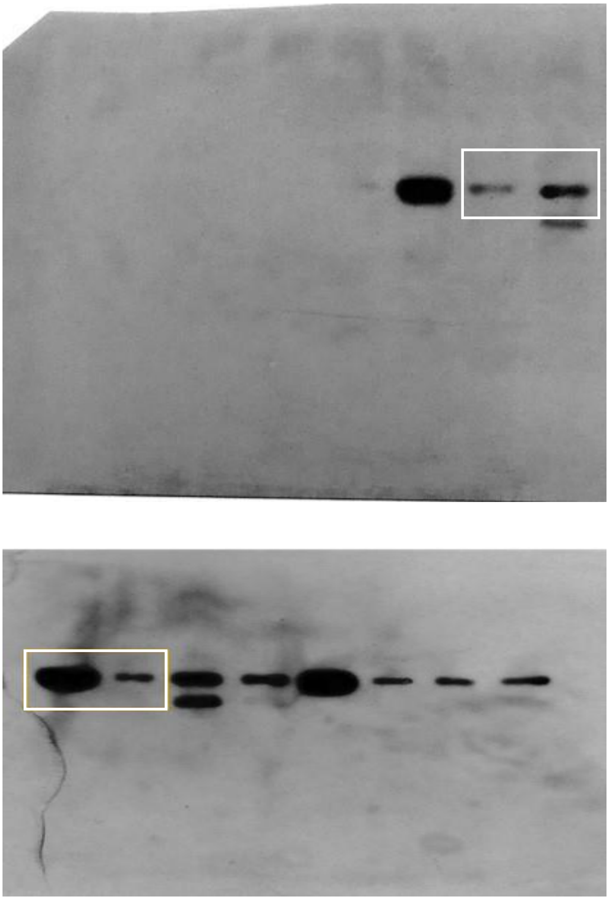

Fig. 3C

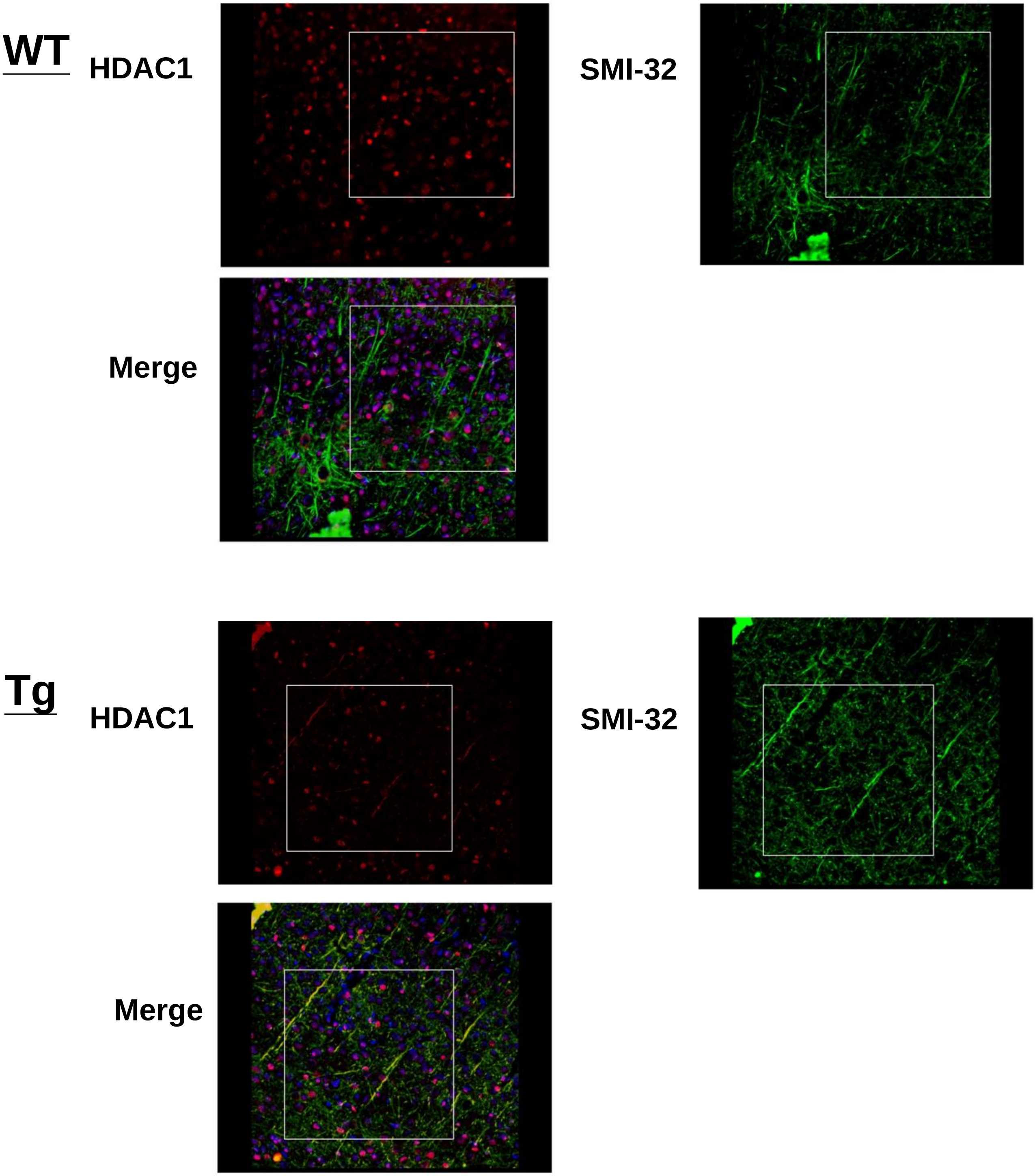

Fig. 3E

Acetyl-H3

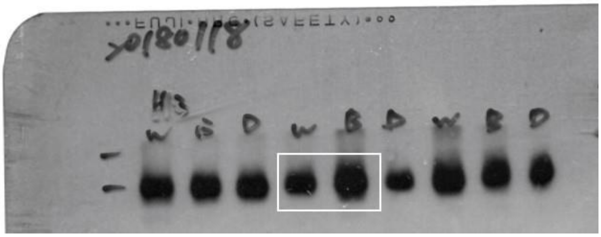

Total-H3

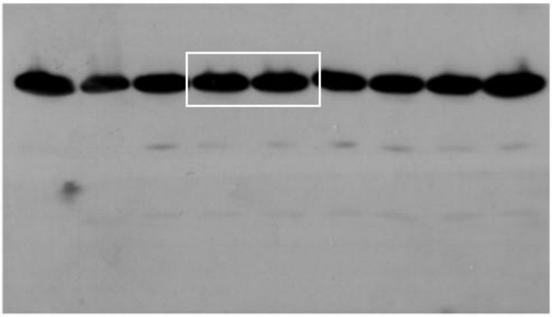

Supplement: Supplementary file 7 — Source Data for Figure 3 [file EMMM-12-e10622-s005.pdf]
