# Supplementary material for: HDAC1 dysregulation induces aberrant cell cycle and DNA damage in progress of TDP‐43 proteinopathies
Source: EMBO Mol Med. 2020 May 25;12(6):e10622. doi: 10.15252/emmm.201910622 (PMC7278561; doi:10.15252/emmm.201910622)

Fig. 4A-1

Tg 1m

TDP-43

HDAC1

Merge

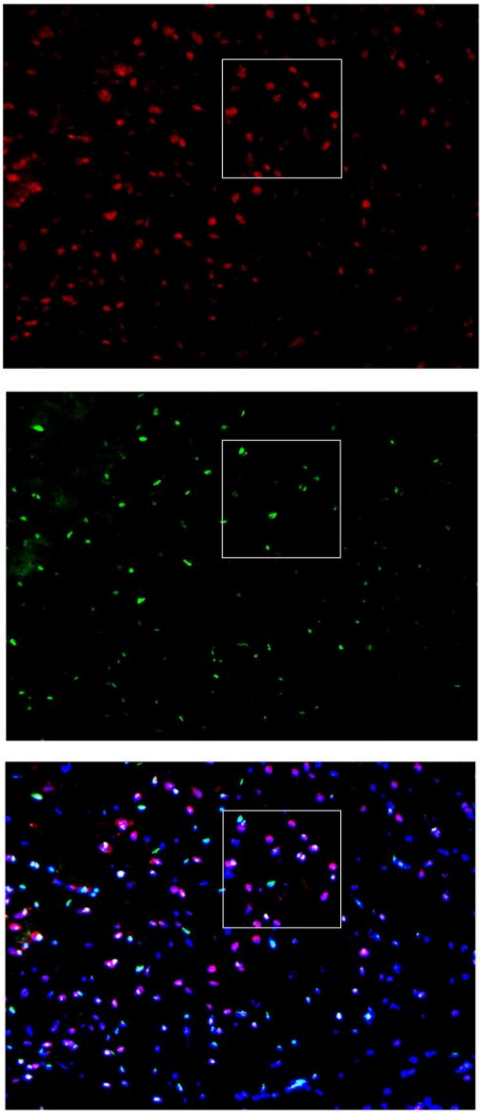

Fig. 4A-2

Tg 6m

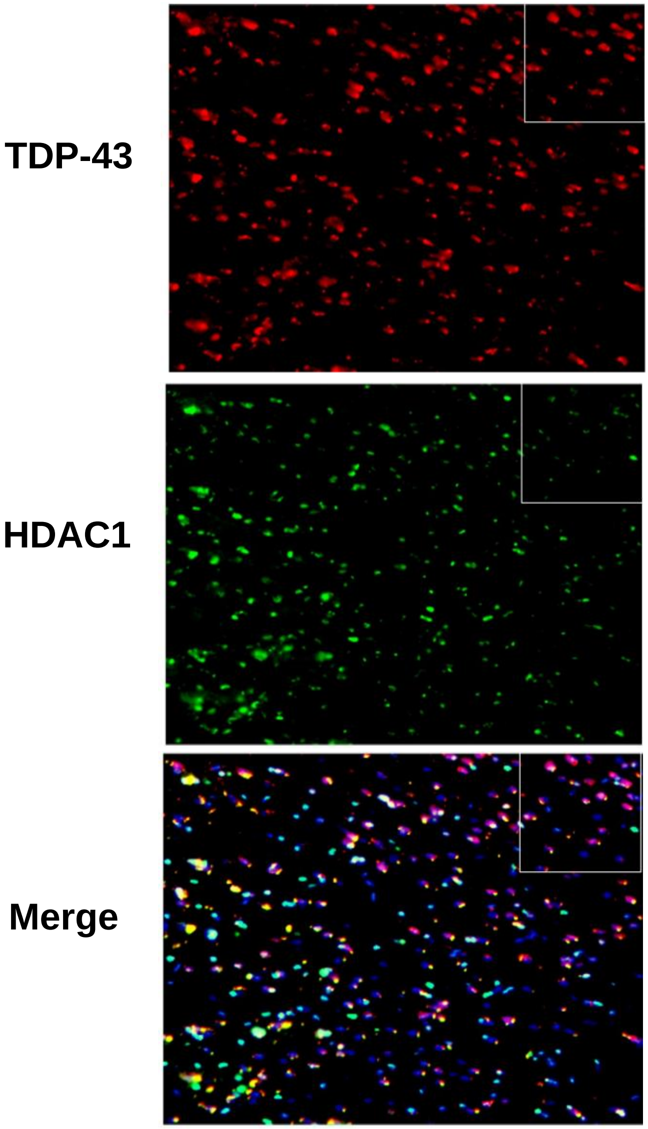

Fig. 4A-3

Tg 12m

TDP-43

HDAC1

Merge

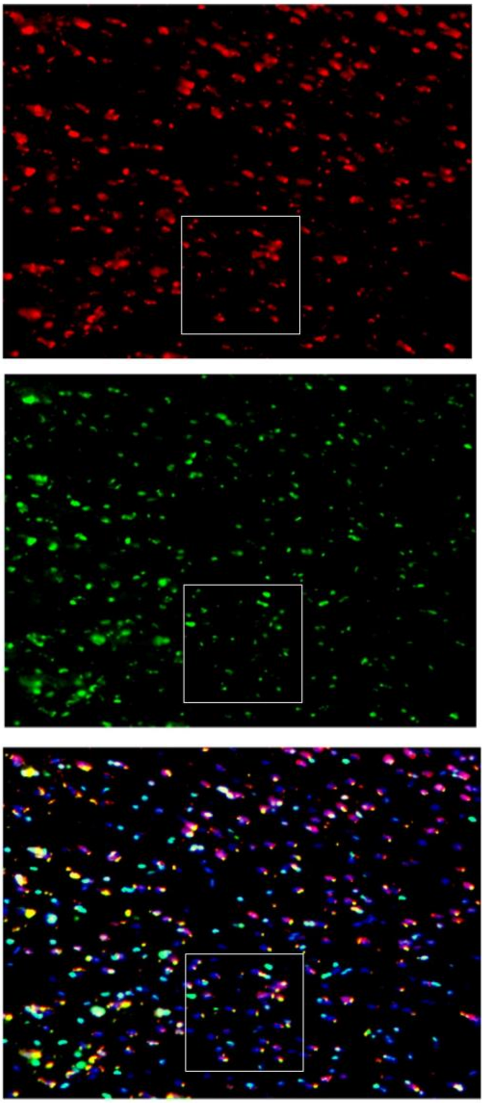

Fig. 4A-4

WT 12m

TDP-43

HDAC1

Merge

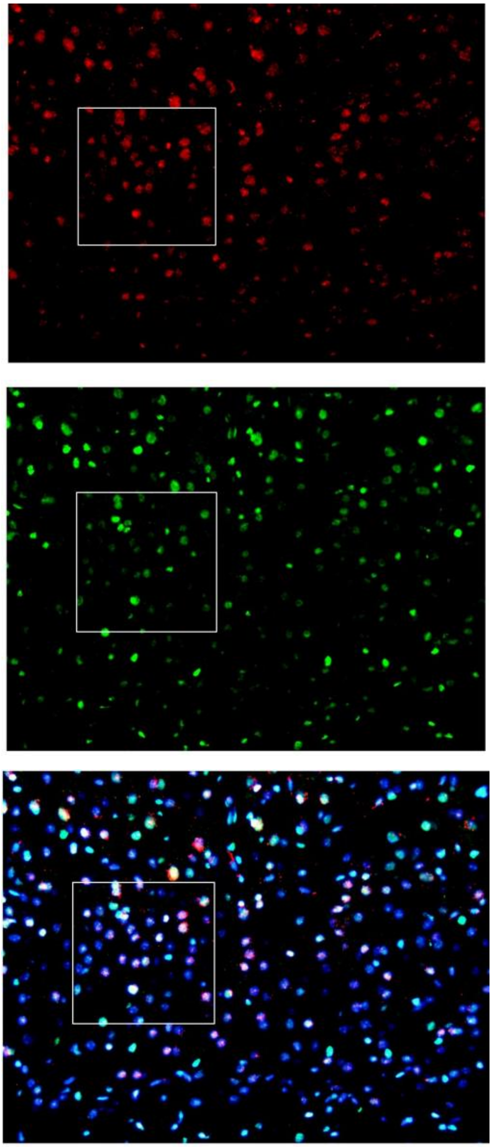

Fig. 4B

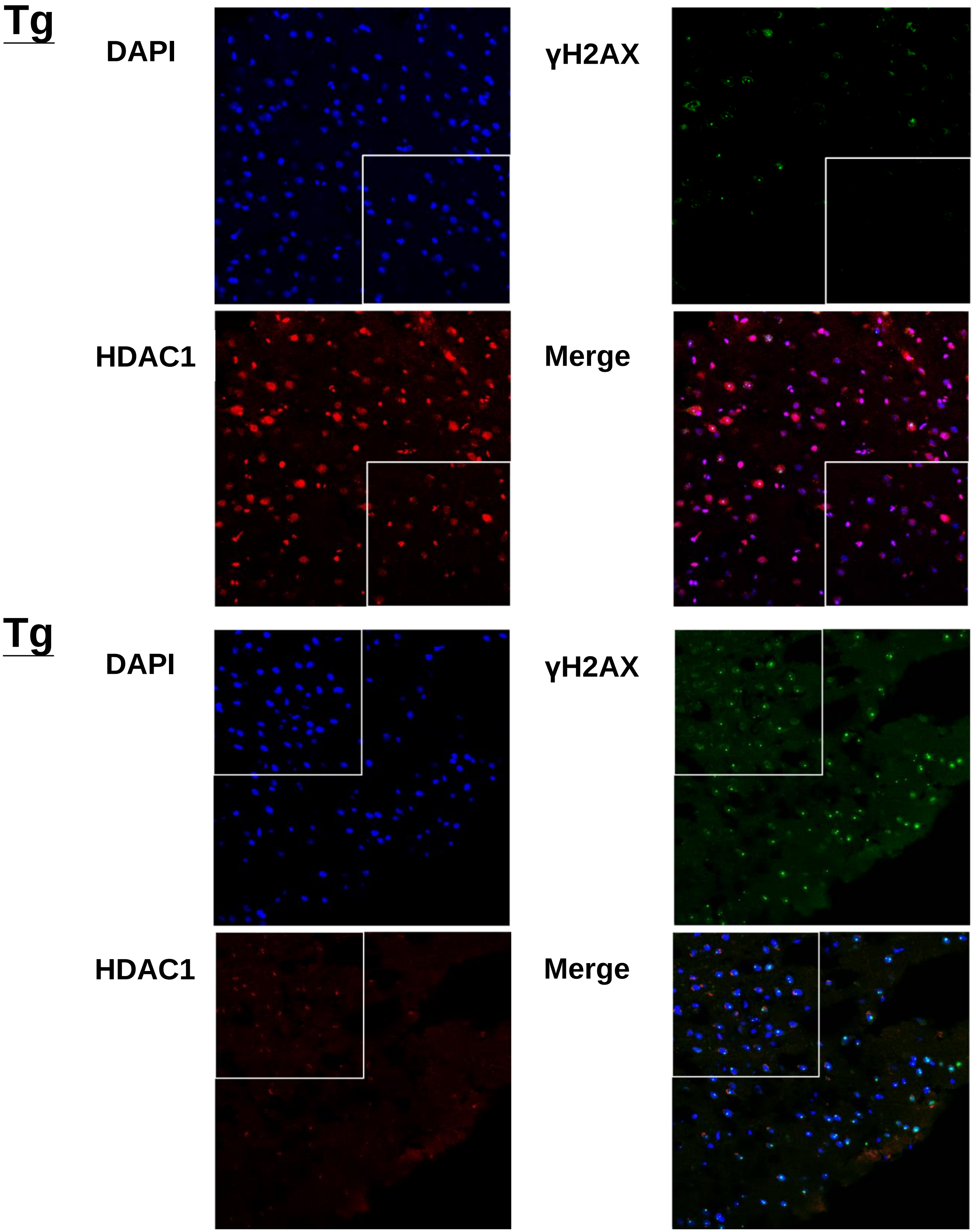

Supplement: Supplementary file 8 — Source Data for Figure 4 [file EMMM-12-e10622-s006.pdf]
