# Supplementary material for: HDAC1 dysregulation induces aberrant cell cycle and DNA damage in progress of TDP‐43 proteinopathies
Source: EMBO Mol Med. 2020 May 25;12(6):e10622. doi: 10.15252/emmm.201910622 (PMC7278561; doi:10.15252/emmm.201910622)

Fig. 5A

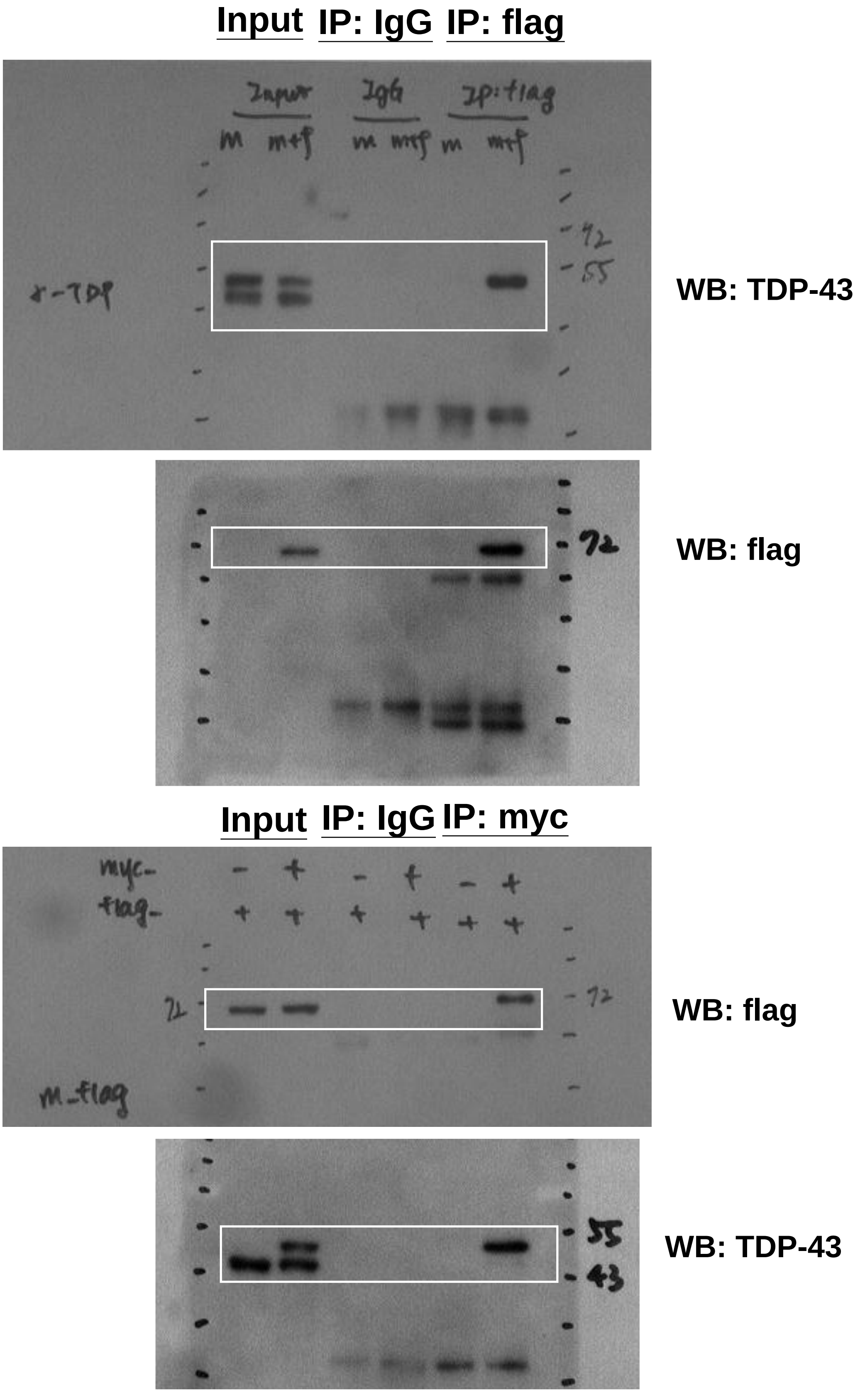

Input
IP: IgG
IP: flag
WB: TDP-43
WB: flag
Input
IP: IgG
IP: myc
WB: flag
WB: TDP-43

Fig. 5B

Input

WB: flag

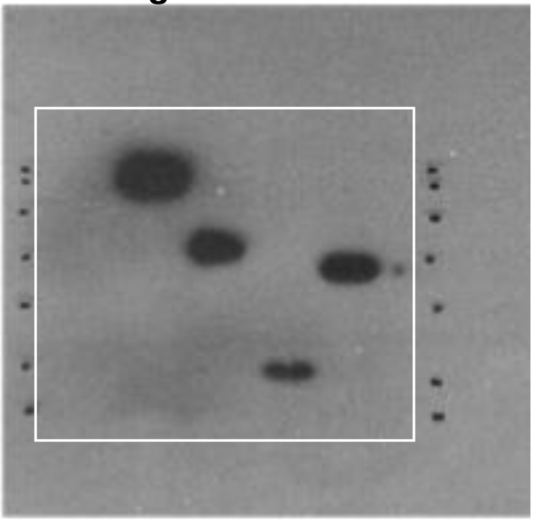

IP: flag

WB:TDP-43

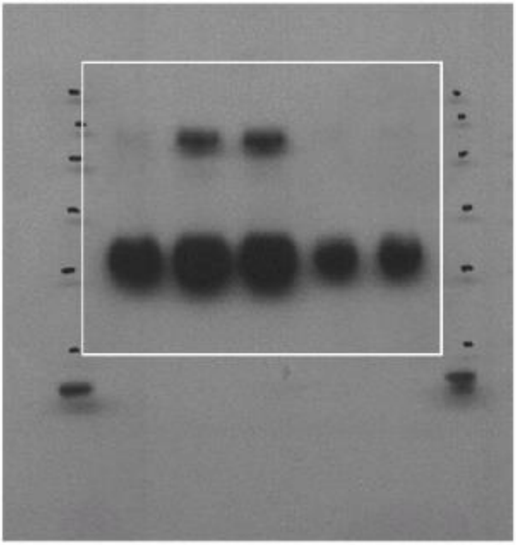

Fig. 5C

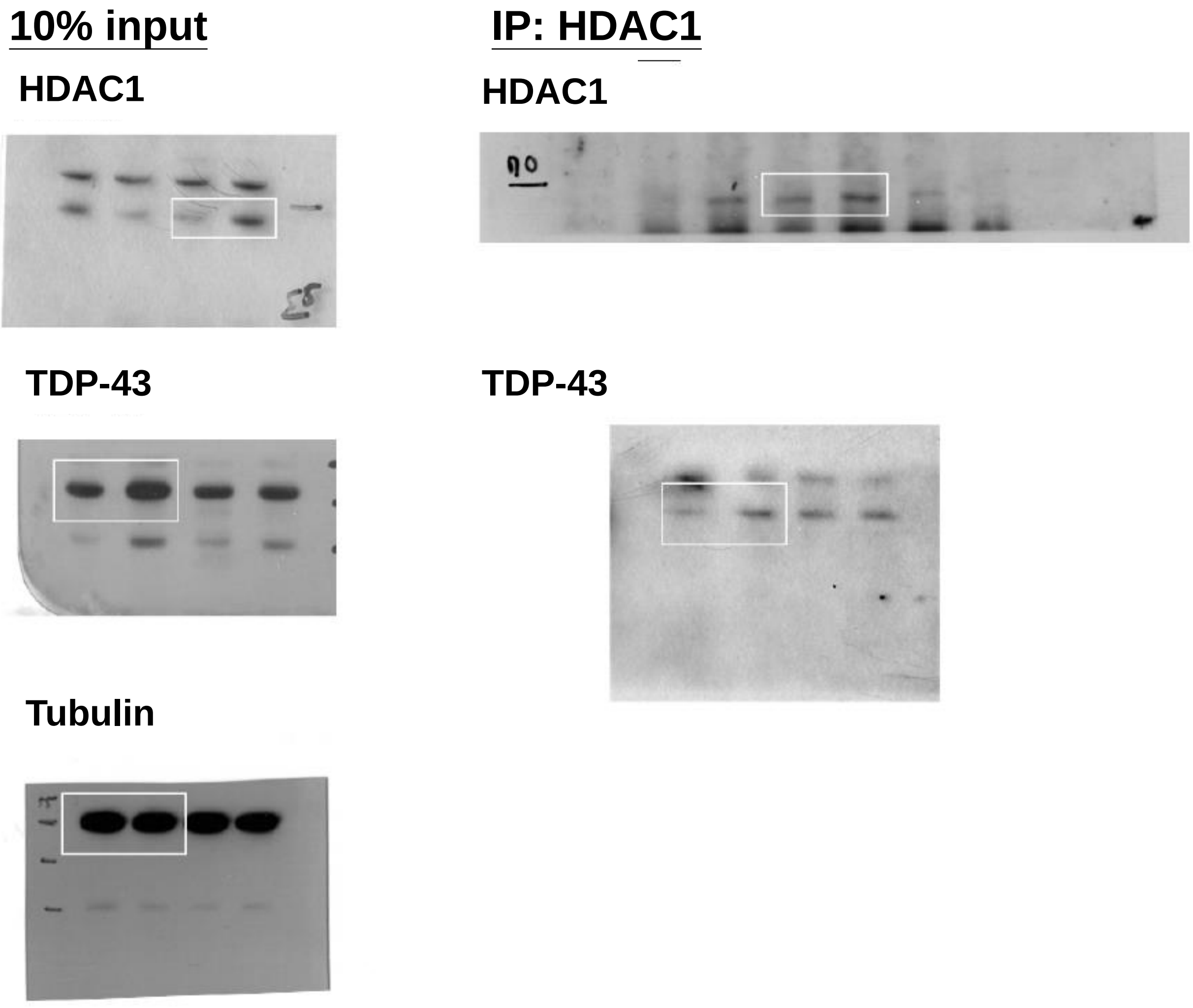

Fig. 5D

HDAC1

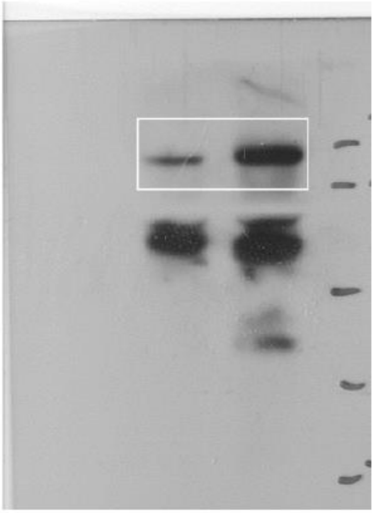

TDP-43

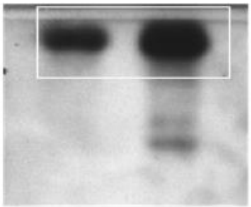

Supplement: Supplementary file 9 — Source Data for Figure 5 [file EMMM-12-e10622-s007.pdf]
