# Supplementary material for: HDAC1 dysregulation induces aberrant cell cycle and DNA damage in progress of TDP‐43 proteinopathies
Source: EMBO Mol Med. 2020 May 25;12(6):e10622. doi: 10.15252/emmm.201910622 (PMC7278561; doi:10.15252/emmm.201910622)
Supplement: Supplementary file 10 — Source Data for Figure 7 [file EMMM-12-e10622-s008.pdf]

Fig. 7B

Acetyl-H3

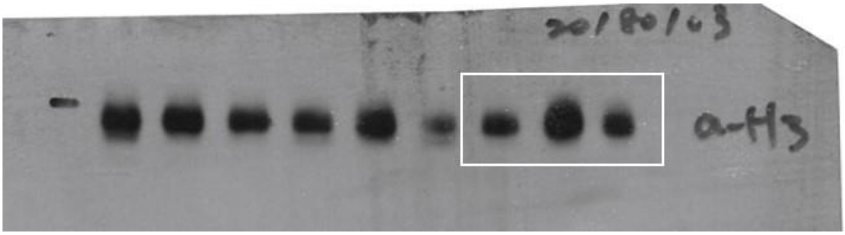

Total-H3

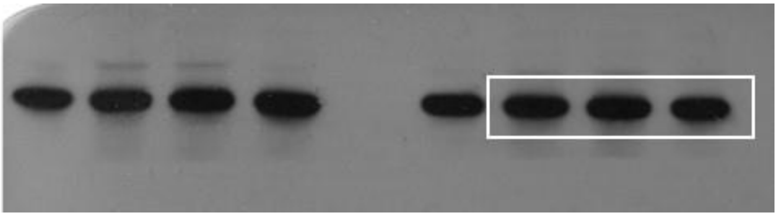

Fig. 7C

Nuclear HDAC1

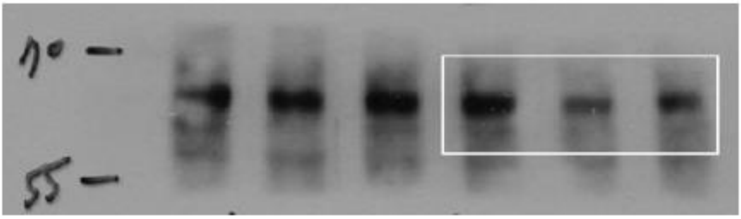

Lamin A

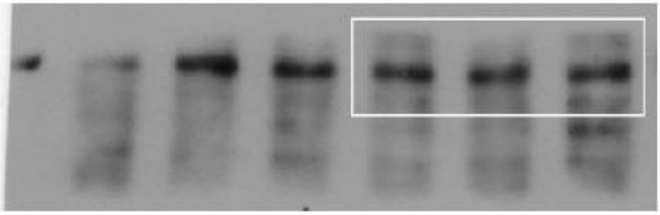

Fig. 7D

E2F1

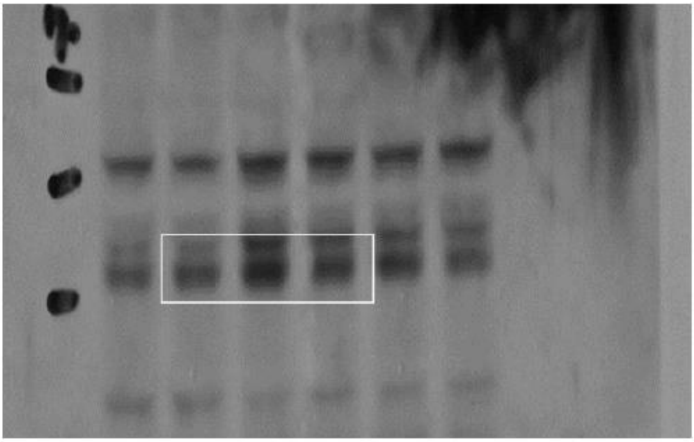

PCNA

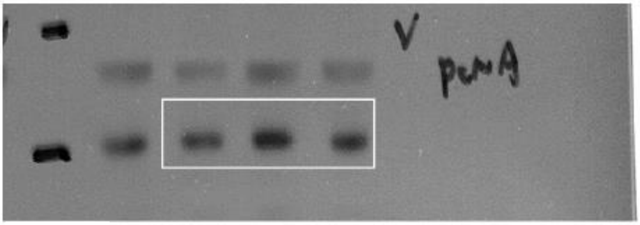

p21

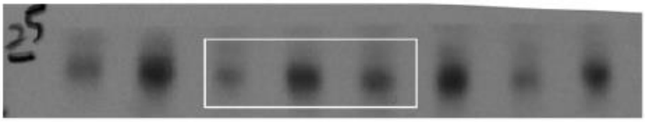

γH2AX

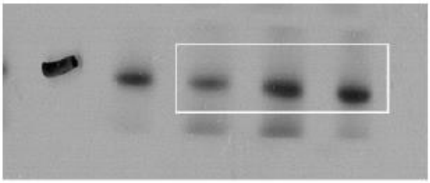

Tubulin

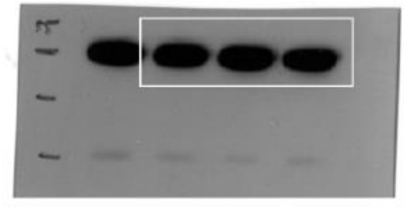

Fig. 7E

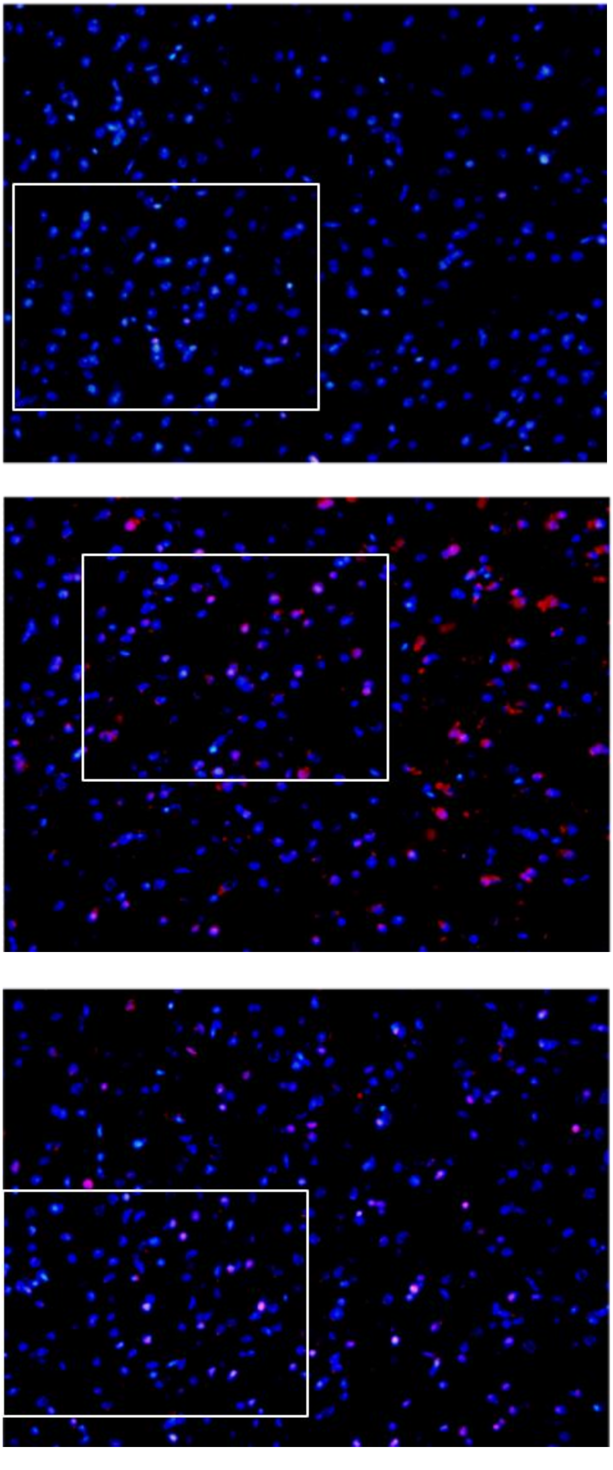

WT
+
Vehicle

Tg
+
Vehicle

Tg
+
5104434

Fig. 7F

WT
+
Vehicle

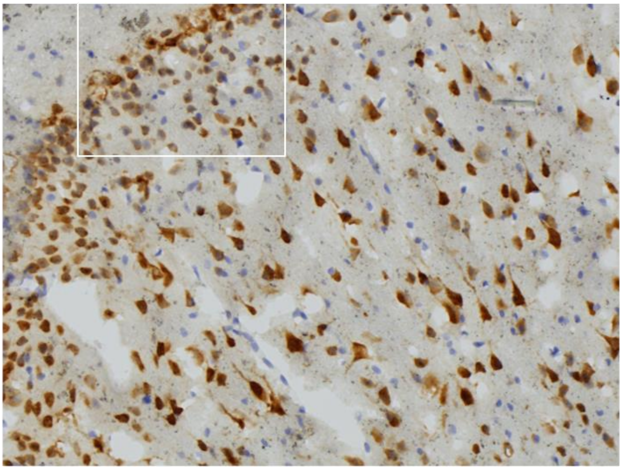

Tg
+
Vehicle

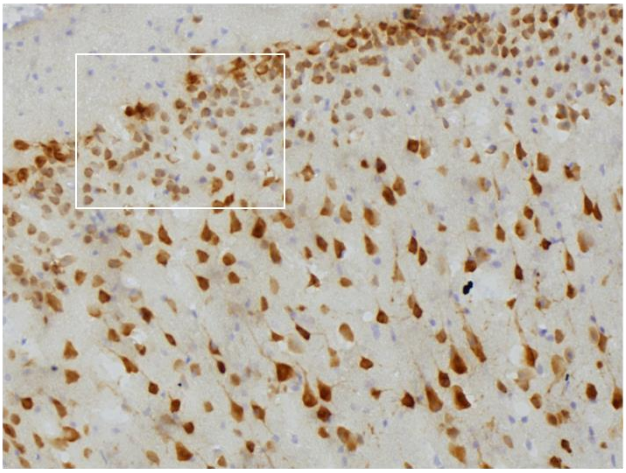

Tg
+
5104434

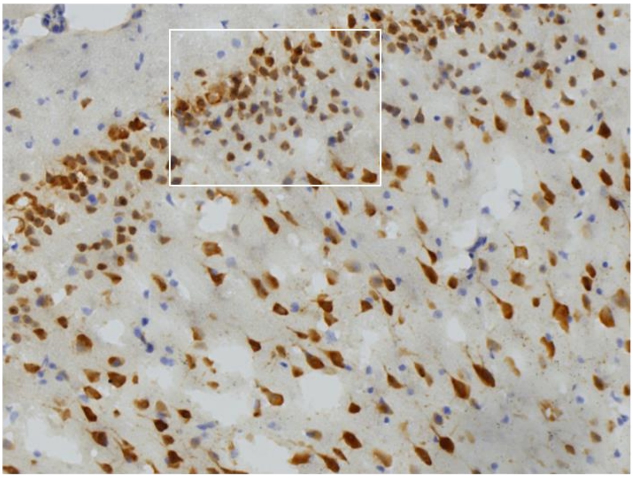

Fig. 7G

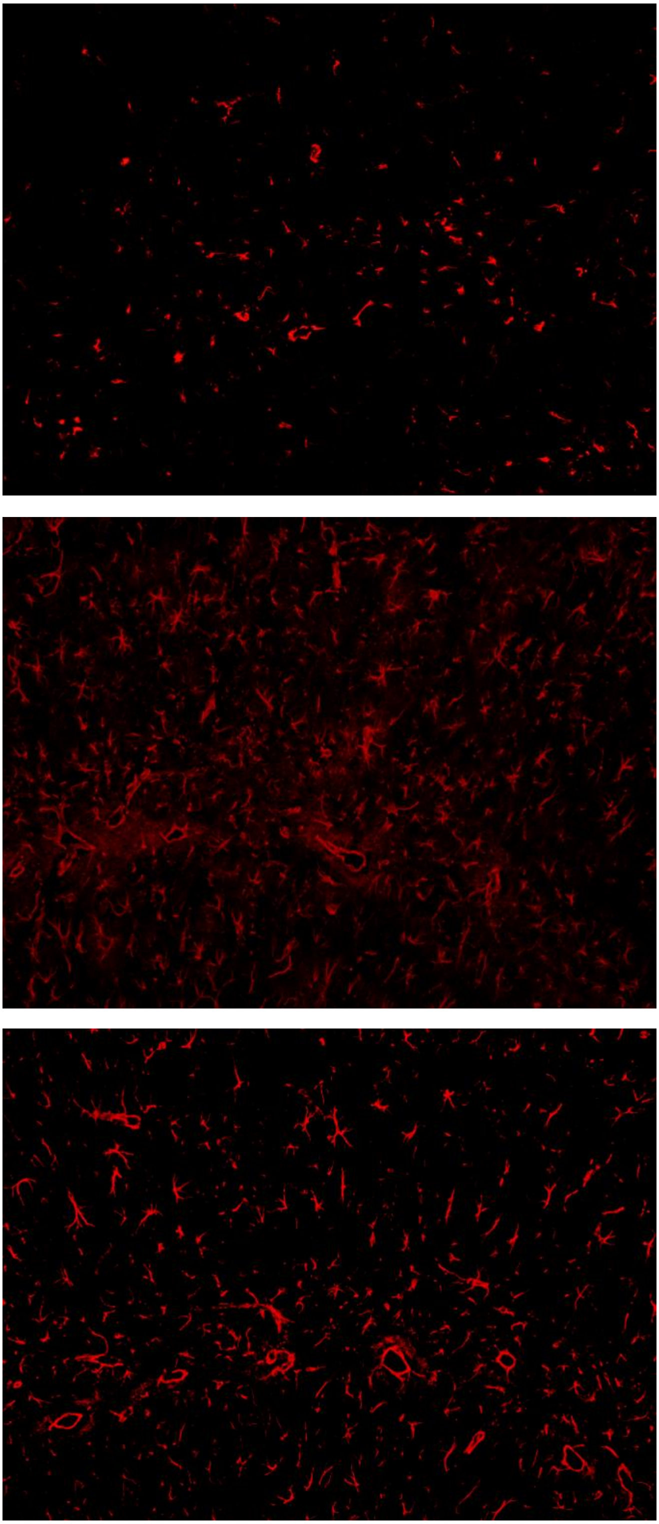

WT
+
Vehicle

Tg
+
Vehicle

Tg
+
5104434
